# Unraveling NO Production in N_2_–O_2_ Plasmas with 0D Kinetic Modeling and Experimental Validation

**DOI:** 10.1021/acs.jpca.4c03323

**Published:** 2024-08-19

**Authors:** Tiago Silva, Susanta Bera, Carlos D. Pintassilgo, Anja Herrmann, Stefan Welzel, Mihalis N. Tsampas, Mauritius C. M. van de Sanden, Luís L. Alves, Vasco Guerra

**Affiliations:** † Instituto de Plasmas e Fusão Nuclear (IPFN), Instituto Superior Técnico, Universidade de Lisboa, 1049-001 Lisboa, Portugal; ‡ Dutch Institute for Fundamental Energy Research (DIFFER), 5612 AJ Eindhoven, the Netherlands; § Departamento de Engenharia Física, Faculdade de Engenharia, Universidade do Porto, 4200-465 Porto, Portugal; ∥ Eindhoven Institute for Renewable Energy Systems (EIRES), Eindhoven University of Technology (TU.e), 5600 MB Eindhoven, the Netherlands; # AFS Entwicklungs + Vertriebs GmbH, Von-Holzapfel-Str. 10, 86497 Horgau, Germany

## Abstract

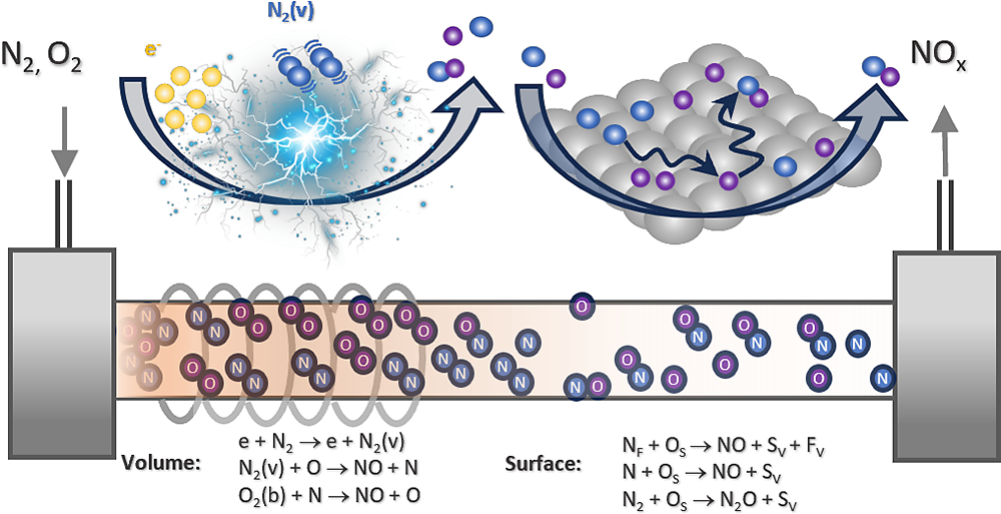

This work presents a detailed investigation aimed at understanding the key mechanisms governing nitric oxide (NO) production in N_2_–O_2_ discharges by systematically comparing experimental results to modeling data. The experimental phase capitalizes on radiofrequency (13.56 MHz) discharges, sustained at 5 mbar pressure conditions, featuring varying concentrations of oxygen, ranging from pure N_2_ plasma to air-like mixtures. On the modeling front, we adopt an integrated approach that combines the solution of the Boltzmann equation for electrons with a system of rate balance equations for heavy species. To account for ground state NO(X) generation at the reactor wall, we combine the volume chemistry with a mesoscopic description of the surface, taking into account adsorption sites and various elementary surface phenomena. Comparisons between experiments and modeling demonstrate very good agreement, extending beyond NO(X) formation to encompass other species in the plasma such as N_2_O(X) and atomic nitrogen N(^4^S). Noteworthy findings include (i) the pivotal role of surface mechanisms in NO(X) production, particularly at low oxygen content values; (ii) the significance of accurately describing the postdischarge phase, where depletion of plasma species occurs at different time scales (millisecond range); and (iii) the importance of vibrationally and electronically excited states (e.g., O_2_(b)) in elucidating NO(X) formation dynamics, crucial for unraveling reaction pathways and energy transfer processes. This work makes an important step toward formulating a comprehensive reaction mechanism for N_2_ and N_2_–O_2_ plasmas applied to nitrogen fixation, covering both volume and surface mechanisms, and lays a robust foundation for future research on plasma-based NO(X) production, particularly in the presence of catalysts.

## 1 Introduction

The shift from fossil fuel reliance to renewable energy integration has become an imperative endeavor, primarily driven by the urgent need to address the challenges posed by climate change. Among the various strategies aimed at addressing this issue, the Power-to-X (P2X) approach has emerged as crucial in the goal to reduce carbon emissions^1^ and mitigate power fluctuations resulting from the intermittency of renewable energy sources.^2^ In the realm of P2X strategies, renewable electricity serves as a pivotal catalyst for the conversion of elemental molecules, including CO_2_ and N_2_, into high-energy compounds and invaluable chemical feedstock. The conversion of CO_2_ is often associated with waste recycling and the generation of hydrocarbon-based neutral fuels through the Fischer–Tropsch synthesis process,^3,4^ while N_2_ conversion is closely linked to nitrogen fixation and production of nitric acid (HNO_3_), which is a crucial source of nitrate utilized in the formulation of plant fertilizers.^5^ These pathways hold paramount significance in addressing the escalating global demand for sustainable energy solutions and fostering environmentally conscious agricultural practices in the face of the world’s rapidly expanding population.

To address these issues, the plasma research community has actively explored nonthermal plasma-based methodologies for gas conversion, with a specific focus on CO_2_ conversion and nitrogen fixation.^6^ Nonthermal plasmas provide a unique advantage by establishing an environment where highly energetic electrons coexist with cold gas, facilitating significant energy transfer crucial for molecular conversion. This characteristic has been extensively documented and investigated for both CO_2_^7−9^ and N_2_^7,8,10,11^ molecules. Moreover, plasma-based systems operate intermittently and instantaneously without requiring preheating or prolonged stabilization, functioning on a subsecond time scale. This is crucial for electricity-driven processes relying on renewable energy sources. A comprehensive study conducted by van Rooij et al.^12^ has delineated a practical framework for the integration of renewable power with plasma-based reactors in the chemical industry, illuminating both the potential and the existing issues of the technology. The study particularly emphasized the challenge of improving energy efficiencies in CO_2_ conversion while highlighting the critical role of separation processes, notably in purifying CO from oxygen.

Concerning scientific research dedicated to plasma-based CO_2_ conversion, Pietanza et al.^13^ provides a comprehensive overview of the latest advancements in nonequilibrium CO_2_ plasma kinetics, synthesizing insights from experimental studies, theoretical analyses, and modeling endeavors. Following the trajectory of many works published on this topic, research efforts are now very much directed toward integrating plasma reactors with separation membrane technology, using either mixed ionic electron conducting materials^14,15^ or solid oxide electrolysis cells.^16^ These endeavors respond to the necessity of separating decomposition products for renewable synthetic fuels and value-added chemical production on an industrial scale. Separation challenges in the plasma environment have also been recently explored under the context of space exploration activities, particularly targeted at In Situ Resource Utilization (ISRU) on Mars. Guerra et al.^17^ addressed the possibility of decomposing CO_2_ under Martian conditions for oxygen production, while proposing the use of separated oxygen and nitrogen (based on mixed ionic electron conducting and ion-conducting membranes) for the production of life support commodities and fertilizers for agriculture. Other efforts aimed at developing plasma-based space applications, as highlighted in recent papers by Engeling and Gott,^18^ Kelly et al.,^19^ and McKinney et al.,^20^ also underscore the significance of gas separation in the context of ISRU.

Regarding scientific research focused on plasma-based nitrogen fixation, a substantial body of experimental work has unfolded in recent years. These investigations encompass a variety of reactor configurations and utilize diverse plasma diagnostics, all aimed at probing the crucial factors that impact the efficiency of NO production. For instance, Abdelaziz et al.^21^ used Fourier transform infrared spectrometer (FTIR) absorbance to study the formation of NO in Spark discharges while striving to reduce energy costs through modifications of reactor geometry. Bahnamiri et al.^22,23^ used FTIR and optical emission spectroscopy in pulsed microwave discharges to measure NO production while studying the effects of specific energy input, pulse frequency, gas flow fraction, gas admixture, and gas flow rate. Pipa et al.^24^ conducted measurements of NO production in radio atmospheric pressure plasma jets using Tunable Diode Laser Absorption Spectroscopy. Hubner et al.^25^ studied the formation of NO, NO_2_, and N_2_O in low-pressure DC plasmas through quantum cascade laser spectroscopy. Patel et al.^26^ investigated NO production within a plasma environment by coupling an inductive RF coil with a solid oxide electrolyzer to generate a stream of H_2_ and O^2–^. Ma et al.^27^ utilized an inductive RF coil to produce NO while scrutinizing the impact of catalysts under various amounts of oxygen content. In a related investigation, Bayer et al.^28^ examined pathways and time scales pertinent to enhancing plasma-assisted N_2_–O_2_ reactions over Ag catalytic surfaces. Various authors have also explored alternative and promising strategies using warm discharges (e.g., rotating gliding arc plasmas) capable of enhancing thermal activation pathways while improving NO yield with increasing pressure and gas quenching methods.^29−31^ A comparative analysis of energy costs for plasma-based NO production in different reactors can be found in the literature.^21−23^ The most promising outcomes in terms of yield and energy efficiency for NO plasma-based production were reported by Rusanov et al.^22,32^ through microwave discharges sustained at low pressure (roughly 10 mbar), reporting an energy cost of 0.29 MJ/mol for a 14% of NO produced from N_2_–O_2_ gas mixtures.

Despite extensive research dedicated to nitrogen fixation in N_2_–O_2_ plasmas, the mechanisms leading to the formation of NO molecules as a result of surface mechanisms remain less thoroughly understood compared with NO production in the discharge volume. This specific challenge has garnered attention in earlier studies, with Hubner et al.^25^ and Pintassilgo et al.^33^ highlighting the potential for NO production through surface-catalyzed recombination in the plasma’s afterglow. This observation finds support in the surface investigations conducted by Marinov et al.^34^ Addressing this issue requires a comprehensive understanding of the complex interplay between gas-phase chemistry within a plasma reactor and the processes of particle loss and formation occurring on reactor surfaces. Indeed, while a well-understood and established kinetic scheme for N_2_–O_2_ plasmas have been developed (as recently reviewed by Guerra et al.^35^), unresolved questions persist regarding volume–surface interactions. In connection with this aspect, recent findings have emerged in the work by Meyer et al.,^36^ elucidating novel phenomena linking O_3_ production to surface mechanisms within N_2_–O_2_ plasmas. These authors considered the adsorption and recombination of O and N atoms and desorption of O_2_ and N_2_ and NO_*x*_ reactions, shedding light on the intricate interplay between gas-phase and surface processes in N_2_–O_2_ plasmas.

To enhance our understanding of nitrogen fixation, particularly concerning surface mechanisms taking place in the plasma reactor, we have developed a self-consistent model for N_2_–O_2_ discharges that combines volume kinetics with surface phenomena leading to NO production. This effort builds upon extensive modeling studies conducted by the Portuguese N-PRiME team at the Institute for Plasma and Nuclear Fusion (IPFN) over several years. Previous studies were focused on (i) analyzing the temporal evolution of heavy species during the active phase and the afterglow of plasma pulses,^37^ (ii) examining power transfer to gas heating in N_2_–O_2_ plasmas,^38^ (iii) investigating the dependence of UV emission intensity on the electronically radiative states of NO,^39^ and (iv) describing coupled electron and heavy-particle kinetics and the role of vibrationally excited N_2_ on NO formation through Zeldovich mechanisms.^35,40^ For the description of surface kinetics in N_2_–O_2_ discharges, this research benefits from a collaborative partnership with the Institute for Fundamental Energy Research (DIFFER) in The Netherlands, enabling a synergistic exchange of modeling knowledge and experimental expertise. This research capitalizes on recent measurements conducted at DIFFER, demonstrating the potential of an inductive coil connected to an RF generator for NO production in the presence of catalysts, thus underscoring the critical role of surface mechanisms in nitrogen fixation.^26,27^

The objectives of this collaborative investigation are 2-fold. First, and aligned with the development of a self-consistent model for N_2_–O_2_ discharges, we aim to achieve validation in terms of NO production through direct comparison with experimental data and to establish a comprehensive framework where interactions between plasma and catalysts can be fully explored. Second, we aim to lay the groundwork for constructing a comprehensive reaction mechanism for N_2_–O_2_ systems, mirroring recent advancements in CO_2_^41^ and O_2_^42^ discharges. Achieving this goal involves the systematic modeling of pure N_2_ and N_2_–O_2_ discharges across a wide range of pressure and power conditions. This requires validating physical mechanisms and reaction rate coefficients to accurately describe experimental data obtained under controlled and homogeneous conditions, such as those typically obtained in DC glow discharges.^41−44^

The structure of this paper is as follows: Section 2 provides a comprehensive description of the experimental setup that forms the foundation for the data analyzed in this work, elucidating the employed reactor and the diagnostics utilized to measure various plasma species. Section 3 offers a thorough description of the model, with subsections dedicated to crucial aspects such as volume kinetics, surface kinetics, and plasma afterglow. Throughout this section, we establish connections between the existing literature and the novel contributions put forward in this work. Section 4 presents the results obtained, focusing on comparative analysis between the modeling results and the experimental data. Finally, in Section 5, we conclude our work and provide perspectives and avenues for future research related to plasma-based nitrogen fixation.

## 2 Experimental Setup

**Figure 1:**
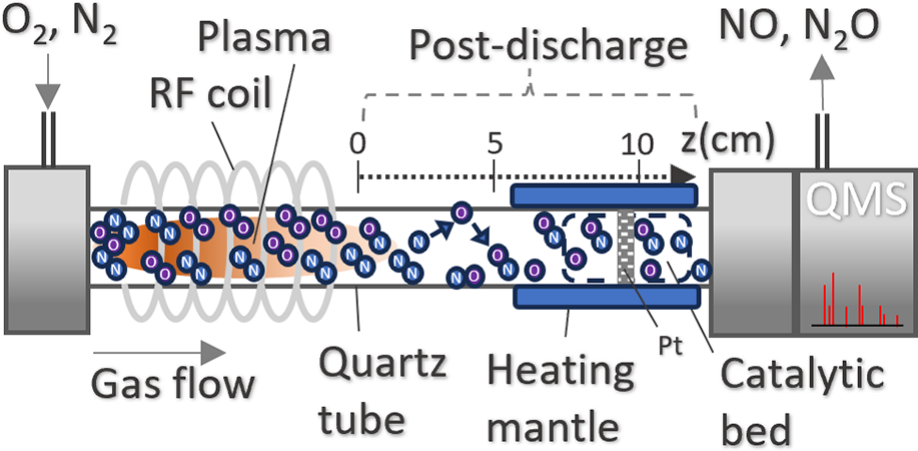
Schematic representation of the RF plasma utilized for the conversion of N_2_ and O_2_ (introduced in the gas inlet) into NO and N_2_O gas fractions detected in the gas outlet. System comprises both plasma and postdischarge regions, with the latter accommodating a catalytic converter. In the catalytic mode, a porous Pt film is coated on the end of a nonporous, capped yttria-stabilized zirconia (YSZ) tube. Marked distances are illustrative, representing typical dimensions separating the plasma and catalytic regions.

The experimental configuration, illustrated in Figure 1, incorporates an inductive coil connected to a 13.56 MHz RF generator, leading to a 14 cm long plasma confined within a quartz tube. Various diagnostic tools complement the experiment, including a Quadrupole Mass Spectrometer (QMS) HAL 201RC for detecting species densities, namely, NO and N_2_O, in the system effluent, a thermocouple probe for gas temperature detection, a Langmuir double probe for characterizing electron temperature and electron density, and catalytic probes coupled with an optical emission spectroscopy (OES) setup (Princeton Instruments Acton SpectraPro SP-2750 spectrometer with a PI-MAX 3 camera) for measuring nitrogen atom density. While maintaining a gas pressure at 5 mbar, the plasma reactor operates with a power input ranging from 40 to 100 W and a total gas flow rate of 100 sccm. As depicted in Figure 1, the plasma is coupled to a catalytic stage featuring Pt catalyst particles on a Yttria-Stabilized Zirconia (YSZ) support, surrounded by a heating mantle, capable of heating the system to approximately 870 K.

**Figure 2:**
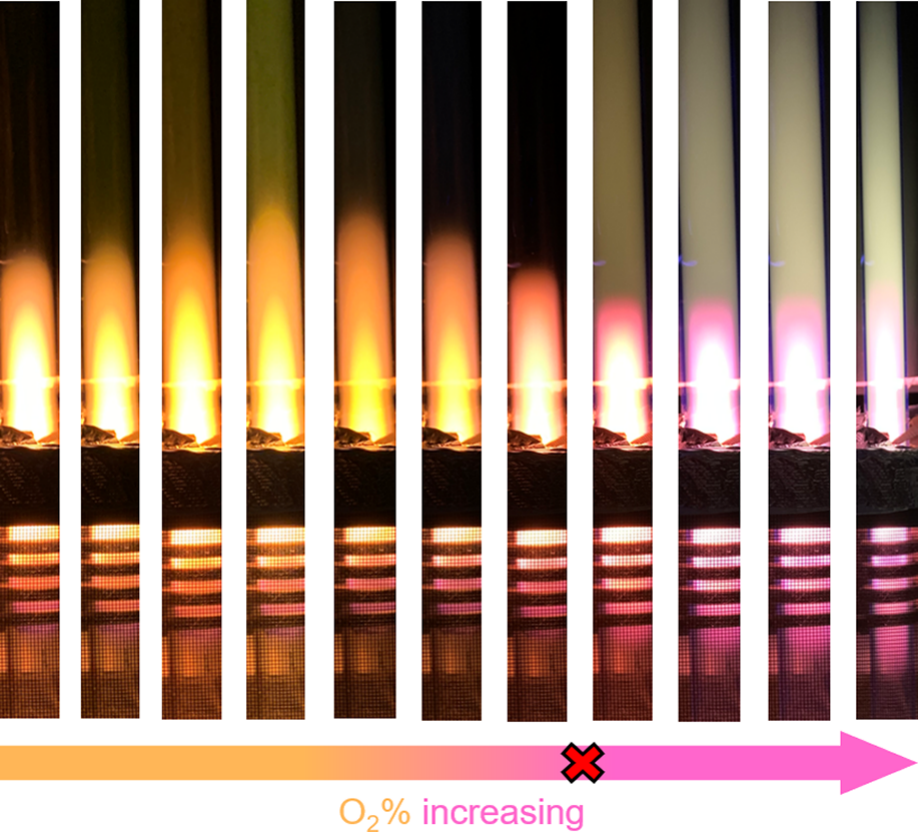
Photographs of the N_2_–O_2_ discharges with plasma lengths of approximately 14 cm long, sustained for different oxygen content values. First figure on the left refers to an oxygen content of 10^–4^, which corresponds to a nearly pure N_2_ environment, while the figure on the right resembles air-like mixtures (oxygen content of 0.3). X mark (shown at 1% of the oxygen content) represents a shift from an environment characterized by nitrogen (N_2_) to one where O_2_ begins to exert influence.

The experimental influence of the Pt catalyst on NO production is shown and discussed below in Section 2.1. It is important to note that, in this work, we have prioritized the analysis of experimental conditions undertaken in the absence of catalysts or a heating mantle. Specifically, our emphasis revolves around understanding the impact of different oxygen content values on NO production. The oxygen content is expressed as [O_2_]/([O_2_] + [N_2_]), where [N_2_] and [O_2_] represent the concentrations of N_2_ and O_2_, respectively. Our investigation spans various oxygen content values, ranging from zero to 0.3, encompassing diverse scenarios from pure N_2_ environments to mixtures closely resembling air-like compositions. Figure 2 visually captures the plasma’s evolution across this spectrum, with the left side dominated by pure N_2_ conditions and the right side depicting mixtures resembling air compositions. The marked transition point in Figure 2 (indicated by an ‘X’) represents the shift from an N_2_-dominated environment to a scenario with a significant introduction of oxygen into the system. For a more in-depth understanding of the experimental setup, the reader is referred to the work of Ma et al.^27^

### 2.1 Experimental Results of Ma et al.^27^

**Figure 3:**
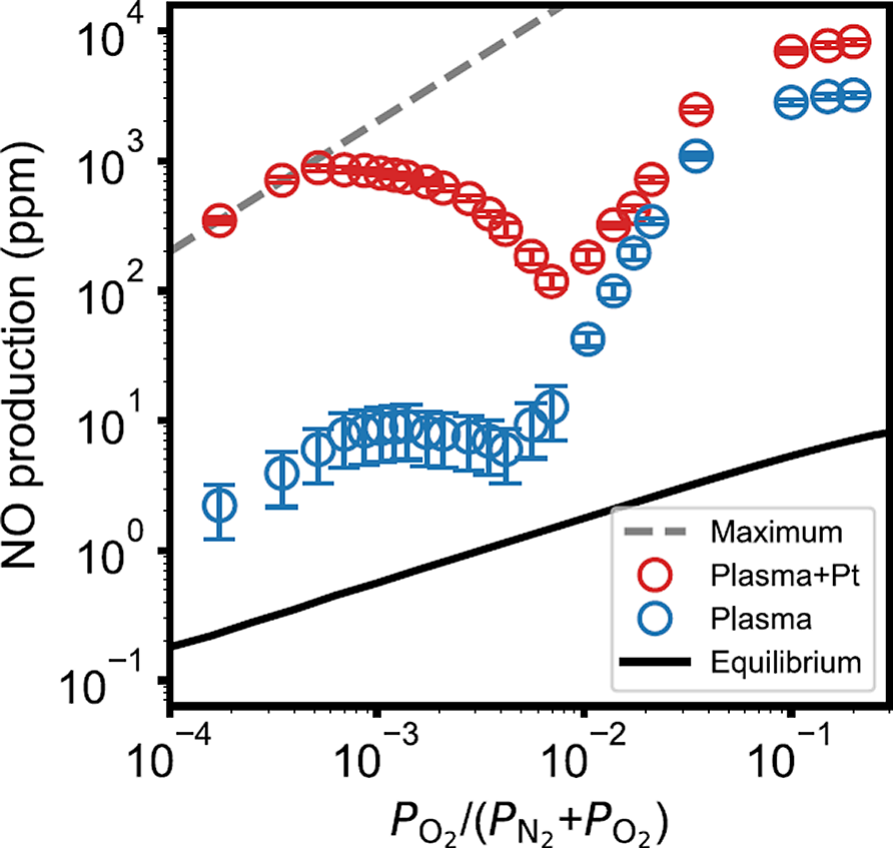
Observed NO production vs normalized partial pressure of O_2_ obtained in Ma et al.^27^ Dashed line is an upper limit, in which all of the O_2_ molecules are converted into NO. These conditions were obtained for a gas pressure of 5 mbar. Figure reproduced from Ma et al.^27^ Available under Creative Commons Attribution 4.0 International License. For information about proper attribution, please see http://creativecommons.org/licenses/by/4.0/.

This study is significantly influenced by the insights initially presented in the work of Ma et al.,^27^ which we briefly summarize in this section. These authors utilized the same radio frequency plasma reactor to investigate NO production in N_2_–O_2_ discharges, both with and without a downstream catalyst (see Figure 3). Their experiments revealed a nonmonotonic increase in NO concentration as a function of the oxygen content in both scenarios. Notably, the Pt catalyst significantly enhanced NO production when the O_2_ fractions were less than 3 × 10^–3^. These findings unequivocally demonstrated that NO production is contingent on both the plasma and Pt catalyst, showing sensitivity to the precise plasma composition. In their study, the authors characterized plasma chemistry using a combination of techniques. They parametrized vibrational heating and compared it to experimentally observed NO production. Surface reactions were further parametrized using data computed through density functional theory. It is also worth mentioning that recent research by Eshtehardi et al.^45^ attempted to interpret these results through modeling and parametric studies. In these studies, the authors considered the profile of vibrational temperatures while imposing a profile of plasma-dissociated species as a function of the oxygen content. In our work, we build upon these insights by employing a self-consistent modeling approach that calculates vibrational populations, dissociation fractions, and NO production. The following sections provide a detailed description of our modeling implementation.

## 3 Kinetic Model

In this section, we present the modeling approach utilized to investigate the N_2_–O_2_ inductively coupled radio frequency plasma discussed in the previous section. Section 3.1 offers an overview of the simulation tool, detailing its main modules and their relevance to the research objectives. In Section 3.2, we describe the specifics of the kinetic mechanisms involved in N_2_–O_2_ discharges, establishing connections between kinetic schemes proposed in the literature and novel elements introduced here. The following sections describe the details related with the surface kinetics, the afterglow region, and numerical implementation in the code workflow.

### 3.1 Overview of the Model

The simulations presented in this study were carried out using the self-consistent LisbOn KInetic (LoKI) numerical simulation tool. LoKI is a global, volume-averaged (0D) kinetic model developed by Tejero et al.^46,47^ and recently upgraded as an open-source tool. The tool has been successfully applied to various plasma systems, including DC glow discharges sustained in O_2_,^42^ CO_2_,^41^ and CO_2_–CH_4_,^48^ inductively coupled discharges sustained in O_2_,^49^ and microwave discharges sustained in N_2_–O_2_.^50^ In summary, LoKI integrates two main calculation blocks: (i) LoKI-B (Boltzmann module), utilized to solve a space-independent form of the electron Boltzmann equation, employing the traditional two-term assumption, and (ii) LoKI-C (Chemistry module), designed to solve a system of 0D rate–balance equations for the heavy species densities in the plasma. In the subsequent paragraphs and subsections, we elaborate on how the input data for each of these modules is managed in the context of this study.

For the Boltzmann module and the calculation of both the electron energy distribution function and electron impact-related rate coefficients, this study incorporates the scattering cross-sections associated with nitrogen and oxygen from the IST-LISBON database of LXCat.^51^ These data sets include (i) twenty-three cross-sections for N_2_, accounting for elastic momentum transfer, excitations to vibrationally excited levels N_2_(X, *v* = 0:10), excitations to various electronic excited states, as well as ionization; (ii) 14 cross-sections for O_2_, including elastic momentum transfer, excitations to vibrationally excited levels O_2_(X, *v* = 1:4), excitations to different electronically excited states, dissociation attachment, and ionization; (iii) four cross-sections for N(^4^S), encompassing elastic momentum transfer, excitation of N(^2^D) and N(^2^P), as well as ionization; and (iv) eight cross-sections for O(^3^P), covering elastic momentum transfer, excitation to several electronically excited states, and ionization. Further details regarding these cross-sections can be found in Guerra et al.^35^

In the chemistry module, this study accounts for the vibrationally excited levels of molecular nitrogen N_2_(X^1^Σ_g_^+^, *v* = 0:59) together with the following electronically excited states of nitrogen N_2_(A^3^Σ_u_^+^, B^3^Π_g_, C^3^Π_u_, a^1^Π_g_, a’^1^Σ_u_^–^, w^1^Δ_u_); ground state and electronically excited molecules of O_2_(X^3^Σ_g_^–^, a^1^Δ_g_, b^1^Σ_g_^+^, Hz), NO(X^2^Π, A^2^Σ^+^, B^2^Σ), NO_2_(X, A), N_2_O(X^1^Σ^+^), O_3_(X), and O_3_^*^; ground state and electronically excited atoms N(^4^S, ^2^D, ^2^P) and O(^3^P, ^1^D, ^1^S); positive ions N^+^(^3^P), N_2_^+^(X^2^Σ_g_^+^, B^2^Σ_u_^+^), N_3_^+^(X), N_4_^+^(X), O^+^(^4^S), O_2_^+^(X), and NO^+^(X); and negative ion O^–^(^2^P). O_2_(Hz) is an effective sum of the O_2_(A′^3^ Δ_u_, A^3^Σ_u_^+^, c^1^Σ_u_^–^) Herzberg states,^52^ and O_3_^*^ represents an effective vibrationally excited ozone level, in line with the approach outlined in Marinov et al.^53^ For the remainder of the text, molecular states will be represented solely by their ground or electronic states, thereby avoiding excessive notation.

Concerning previous works,^35^ note that the current model additionally includes the kinetics of N_2_O(X) and O(^1^S). Further details will be provided in a subsequent subsection regarding the inclusion of these species. It is also important to emphasize that we have neglected the vibrational distribution for O_2_(X). This decision is motivated by considerations of computational efficiency and the relatively less excited vibrational distribution functions of oxygen molecules compared with molecular nitrogen. This approach is aligned with the methodology employed in the work of Pintassilgo et al.^33^ and finds support in the relatively limited population of vibrationally excited O_2_ molecules observed in the calculations by Annusová et al.^49^ Nevertheless, it is important to note that in pure O_2_ discharges, accounting for vibrational distribution becomes significant when characterizing gas temperature due to heating resulting from collisions between oxygen atoms and vibrationally excited oxygen, as demonstrated in Dias et al.^42^ Considering that, in this work, the oxygen fraction relative to nitrogen is below 30%, we anticipate that the contribution of this heating mechanism is negligible compared to other mechanisms involving, for example, the deactivation of vibrationally excited N_2_ with atomic atoms. Additional specifics regarding the gas temperature calculations will be provided later in this subsection.

To gain a deeper understanding of the chemistry module used to describe N_2_–O_2_ discharges, it is valuable to elucidate the distinct gain and loss terms linked to the various species. These terms can be expressed as follows for any species *N*_*i*_:
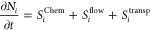
1The term *S*^Chem^ encompasses electron–impact reactions employing rate coefficients obtained from the Boltzmann module as well as chemical reactions among heavy particles, such as vibrational transitions and gas-phase chemistry. Further details regarding the reactions considered in this term be provided in the subsequent section. The term *S*^flow^ includes the creation and destruction resulting from the in-flow and outflow of particles in the reactor. The outflow is computed to ensure the conservation of the total number of atoms within the discharge volume, following the methodology outlined by Sovelas et al.^41^ The term *S*^transp^ accounts for the transport of charged and neutral species to the plasma reactor. Concerning the transport of charged species, we assume that positive ions follow an ambipolar diffusion, in line with the high plasma density limit of ion transport theories.^54^ While the impact of negative ions O^–^(^2^P) is not expected to be significant in N_2_–O_2_ discharges with high nitrogen content,^55^ we also consider their potential effect on the electron density profile, following the insights from Dias et al.^42^ The transport parameters, including mobility and free diffusion coefficients for positive ion species associated with N_2_–O_2_ discharges, are adopted from Coche et al.^50^

To model the transport of neutral species toward the reactor surface, we combine a mesoscopic formulation^56^ with a global approach proposed by Chantry.^57^ On the one hand, in the mesoscopic formulation (further detailed in Section 3.3), the time-evolution of the adsorbed species and adsorption sites is deterministically solved by a set of differential equations. On the other hand, the global approach takes into account two crucial factors: the diffusion time of species from the reactor volume to the wall and the surface deactivation/recombination probability (represented as γ_*i*_) of these species at the reactor wall. In this global approach, the transport source term is described by
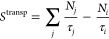
2where τ_*i*_ represents the characteristic transport time for species *i* from the center of the discharge to the wall. The summation in 2 encompasses all species *j* whose deactivation/recombination at the wall contributes to the creation of species *i*. For cylindrical geometry, the characteristic transport is determined according to
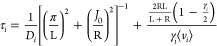
3where R and L correspond to the radius and length of the discharge tube, respectively; *J*_0_ is approximately equal to 2.405 and represents the first zero of the zero-order Bessel function; ⟨*v*_*i*_⟩ represents the thermal velocity of species *i*; *D*_*i*_ is its diffusion coefficient; and γ_*i*_ is the wall recombination probability. In this work, *D*_*i*_ is calculated from the simplified Wilke’s formula.^58,59^ Note that in 3, the limiting cases τ_*i*_^–1^ ≃ γ_*i*_⟨*v*_*i*_⟩/(2R) and τ_*i*_^–1^ ≃ *D*_*i*_(2.405/R)^2^ are recovered when γ_*i*_ ≪ 1 and γ_*i*_ → 1, respectively. Section 3.3 provides detailed insights into the selection of γ_*i*_ values for different plasma species.

Lastly, it is important to note that the chemistry module also solves the thermal model to self-consistently calculate the average gas temperature *T*_g_. For describing the average gas temperature, we assume a constant pressure and consider heat conduction as the primary cooling mechanism within the volume of the reactor. The gas thermal balance equation is then expressed as follows, accounting for a parabolic radial temperature profile:^38^
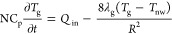
4where *N* represents the gas density, *C*_p_ denotes the specific heat capacity of the gas at constant pressure, *Q*_in_ stands for the total net power per unit volume transferred to gas heating, λ_g_ refers to the thermal conductivity, and *T*_nw_ indicates the gas temperature near the wall of the tube. The relationship between *T*_nw_ and the average gas temperature *T*_g_ has been the subject of investigation in various studies, including experimental campaigns, such as the one conducted by Booth et al.^43^ In light of these studies, we make the additional assumption that in the near-wall region, the thermal balance equation yields equality between the power per unit area flowing outward from the gas/plasma system via conduction and the flow inward in the region near the tube wall via convection, which is dependent on the wall temperature *T*_w_. The value of *T*_w_ is set as a boundary condition at 323 K. Further details can be found in Dias et al.^42^ Input values associated with conductivity, heat capacity, and reaction-dependent energy for gas heating are adopted from the work of Pintassilgo et al.^37,38^

### 3.2 N_2_–O_2_ Kinetic Scheme

For the N_2_–O_2_ kinetic scheme considered in this work, we capitalize from the recently developed reaction mechanism for pure O_2_ discharges (see Tables 1 and 2 in Dias et al.^42^) and the set of reactions concerning pure N_2_ discharges presented in previous works (see Table 2 in Guerra et al.^60^ and Tables 1, 2, 3, 4, and 5 in Guerra et al.^35^). Furthermore, we extend the list of reactions associated with interactions between nitrogen and oxygen (more details below), previously given in the works of Pintassilgo et al.^39^ and Coche et al.^50^ Concerning reactions involving pure N_2_, it is worth mentioning that we assume: (i) electron impact dissociation from all the vibrational excited levels of N_2_ with the same cross-section used for *v* = 0 and (ii) ionization and excitation of electronically excited states (e.g., N_2_(A)) with single energy-loss processes involving only the ground state level N_2_(X, *v* = 0). These assumptions mirror those utilized in previous works^60,61^ and stem from the absence of available cross-sections involving excitation/dissociation from highly excited vibrational levels. The effect of considering a more complex scheme with several transitions involving various vibrational levels (based on Franck–Condon approximations) has been studied in Dyatko et al.^62^ and Loureiro et al.^63^ The analyses of these effects in terms of NO(X) production fall outside the scope of this study. Ongoing efforts dedicated to the validation and improvement of the kinetic scheme of pure N_2_ discharges, mirroring that the advancements recently made in O_2_ studies^42^ will carefully consider these aspects.

**Table 1:** List of Reactions Describing Heavy Species Collisions between Oxygen and Nitrogen Molecules^a^

process	rate coefficient (S.I.)	ref
R1: O_2_(b) + N_2_(X) → O_2_(a) + N_2_(X)	1.7 × 10^–21^ (*T*_g_/300)	(78)
R2: N(^2^D) + O(^3^P) → N(^4^S) + O(^3^P)	1.1 × 10^–18^	(79)
R3: N_2_(A) + O_2_(X) → N_2_(X, *v* = 0) + O_2_(a)	1.29 × 10^–18^	(65)
R4: N_2_(A) + O_2_(X) → N_2_(X, *v* = 0) + 2O(^3^P)	1.63 × 10^–18^ (*T*_g_/300)^0.55^	(55)
R5: N_2_(B) + O_2_(X) → N_2_(X, *v* = 0) + 2O(^3^P)	3 × 10^–16^	(65)
R6: N_2_(C) + O_2_(X) → N_2_(X, *v* = 0) + 2O(^3^P)	3 × 10^–16^	(65)
R7: N_2_(a) + O_2_(X) → N_2_(X, *v* = 0) + 2O(^3^P)	4.3 × 10^–16^	(55)
R8: N_2_(a′) + O_2_(X) → N_2_(X, *v* = 0) + 2O(^3^P)	2.8 × 10^–17^	(80)
R9: N_2_(w) + O_2_(X) → N_2_(X, *v* = 0) + 2O(^3^P)	10^–16^	(81)
R10: N(^4^S) + N(^4^S) + O_2_(X) → N_2_(X, *v* = 0) + O_2_(X)	8.27 × 10^–46^ Exp[500/*T*_g_]	(65)
R11: O(^3^P) + O(^3^P) + N_2_(X) → O_2_(X) + N_2_(X)	2.76 × 10^–46^ Exp[720/*T*_g_]	(65)
R12: O(^3^P) + O_2_(X) + N_2_(X) → O_3_(X) + N_2_(X)	5.7 × 10^–46^ (300/*T*_g_)^2.8^	(39)
**R13**: O(^1^D) + N_2_(X) → O(^3^P) + N_2_(X)	1.8 × 10^–17^ Exp[107/*T*_g_]	(65)

a Rate coefficients are expressed in m^3^ s^–1^ and m^6^ s^–1^ for two-body and three-body reactions, respectively. Gas temperature, *T*_g_, is expressed in K. Mechanisms in **bold** are novel/corrected in comparison to previous studies.

**Table 2:** List of Reactions Describing Heavy Species Collisions Involving NO(X,A,B)^a^

process	rate coefficient (S.I.)	ref
R14: N_2_(X, *v* = 13: 59) + O(^3^P) → NO(X) + O(^3^P)	1.0 × 10^–19^	(35,40)
R15: N_2_(A) + O(^3^P) → NO(X) + N(^2^D)	7 × 10^–18^	(40)
R16: O_2_(X) + N(^4^S) → NO(X) + O(^3^P)	1.1 × 10^–20^*T*_g_ Exp[−3150/*T*_g_]	(65)
R17: O_2_(X) + N(^2^D) → NO(X) + O(^3^P)	1.5 × 10^–18^ (*T*_g_/300)^0.5^	(65)
R18: O_2_(X) + N(^2^D) → NO(X) + O(^1^D)	6 × 10^–18^ (*T*_g_/300)^0.5^	(65)
R19: O_2_(X) + N(^2^P) → NO(X) + O(^3^P)	2.6 × 10^–18^	(65)
R20: O_2_(a) + N(^4^S) → NO(X) + O(^3^P)	2 × 10^–20^ Exp[−600/*T*_g_]	(65)
R21: N(^4^S) + O(^3^P) → NO(A)	1.18 × 10^–23^ (300/*T*_g_)^0.35^	(82)
R22: N(^4^S) + O(^3^P) + N_2_(X) → NO(X) + N_2_(X)	1.76 × 10^–43^ (1/*T*_g_)^0.5^	(65)
R23: N(^4^S) + O(^3^P) + N_2_(X) → NO(A) + N_2_(X)	2.12 × 10^–46^ (300/*T*_g_)^1.24^	(82)
R24: N(^4^S) + O(^3^P) + N_2_(X) → NO(B) + N_2_(X)	3.09 × 10^–46^ (300/*T*_g_)^1.4^	(82)
R25: N(^4^S) + O(^3^P) + O_2_(X) → NO(X) + O_2_(X)	1.76 × 10^–43^ (1/*T*_g_)^0.5^	(65)
R26: N(^4^S) + O(^3^P) + O_2_(X) → NO(B) + O_2_(X)	3.09 × 10^–46^ (300/*T*_g_)^1.4^	(82)
R27: NO(X) + N_2_(A) → NO(A) + N_2_(X, *v* = 0)	6.6 × 10^–17^	(83)
R28: NO(A) + N_2_(A) → NO(X) + N_2_(X)	10^–19^	(39)
R29: NO(A) + O_2_(X) → NO(X) + O_2_(X)	1.5 × 10^–16^	(39)
R30: NO(A) + NO(X) → NO(X) + NO(X)	2 × 10^–16^	(39)
R31: NO(B) + N_2_(X) → NO(X) + N_2_(X)	6.1 × 10^–19^	(39)
R32: NO(B) + O_2_(X) → NO(X) + O_2_(X)	1.5 × 10^–17^	(39)
R33: NO(B) + NO(X) → NO(X) + NO(X)	2 × 10^–16^	(39)
R34: NO(X) + N(^4^S) → N_2_(X, *v* = 3) + O(^3^P)	1.05 × 10^–16^ (*T*_g_)^0.5^	(39)
R35: NO(X) + N(^2^D) → N_2_(X, *v* = 0) + O(^3^P)	6.3 × 10^–17^	(84)
R36: NO(X) + N(^2^P) → N_2_(A) + O(^3^P)	3.4 × 10^–17^	(65)
R37: NO(X) + N_2_(a′) → N(^4^S) + N_2_(X, *v* = 0) + O(^3^P)	3.6 × 10^–16^	(80)
R38: N_2_(B) + NO(X) → N_2_(A) + NO(X)	2.4 × 10^–16^	(65)
R39: O_2_(b) + NO(X) → O_2_(a) + NO(X)	6.0 × 10^–20^	(78)
R40: O_2_(a) + NO(X) → O_2_(X) + NO(X)	2.5 × 10^–23^	(85)
R41: NO(A) → NO(X) + *hv*	4.5 × 10^6^	(50)
R42: NO(B) → NO(X) + *hv*	3.0 × 10^5^	(85)
R43: O(^1^D) + NO(X) → N(^4^S) + O_2_(X)	1.7 × 10^–16^	(65)
**R44**^T^: O_2_(b) + N(^4^S) → NO(X) + O(^3^P)	2.5 × 10^–16^	(64)

a Rate coefficients are expressed in m^3^ s^–1^ and m^6^ s^–1^ for two-body and three-body reactions, respectively. Transition probabilities are expressed in *s*^–1^ for the radiative transitions. Gas temperature, *T*_g_, is expressed in K. Mechanisms in **bold** are novel/corrected in comparison to previous studies. Reactions with superscript T (not included in the default chemistry module) are tested in this work with an upper limit rate coefficient that approximates the gas kinetic rate.

**Table 3:** List of Reactions Describing Heavy Species Collisions Involving NO_2_(X)^a^

process	rate coefficient (S.I.)	ref
R45: NO(X) + O_3_(X) → NO_2_(X) + O_2_(X)	4.3 × 10^–18^ Exp[−1560/*T*_g_]	(65)
R46: NO(X) + O(^3^P) + N_2_(X) → NO_2_(X) + N_2_(X)	10^–43^	(86)
R47: NO(X) + O(^3^P) + N_2_(X) → NO_2_(A) + N_2_(X)	3.7 × 10^–44^	(87)
R48: NO(X) + O(^3^P) + O_2_(X) → NO_2_(X) + O_2_(X)	8.6 × 10^–44^	(88)
R49: NO(X) + O(^3^P) + O_2_(X) → NO_2_(A) + O_2_(X)	3.7 × 10^–44^	(87)
R50: NO_2_(A) + N_2_(X) → NO_2_(X) + N_2_(X)	6 × 10^–17^	(89)
R51: NO_2_(A) + O_2_(X) → NO_2_(X) + O_2_(X)	6 × 10^–17^	(39)
R52: NO_2_(X) + N(^4^S) → N_2_(X, *v* = 0) + O_2_(X)	7 × 10^–19^	(65)
R53: NO_2_(X) + N(^4^S) → N_2_(X, *v* = 0) + 2O(^3^P)	9.1 × 10^–19^	(65)
R54: NO_2_(X) + N(^4^S) → NO(X) + NO(X)	2.3 × 10^–18^	(65)
R55: NO_2_(A) → NO_2_(X) + *hv*	2.5 × 10^4^	(89)
**R56**: NO_2_(X) + O(^3^P) → NO(X) + O_2_(X)	3.26 × 10^–18^*T*_g_^0.18^	(39)

a Rate coefficients are expressed in m^3^ s^–1^ and m^6^ s^–1^ for two-body and three-body reactions, respectively. Transition probabilities are expressed in s^–1^ for radiative transitions. Gas temperature, *T*_g_, is expressed in K. Mechanisms in **bold** are novel/corrected in comparison to previous studies.

**Table 4:** List of Reactions Describing Heavy Species Collisions Involving N_2_O(X)^a^

process	rate coefficient (S.I.)	ref
**R57**: N_2_(X, *v* = 15: 59) + O_2_(a) → N_2_O(X) + O(^1^D)	1.0 × 10^–20^	(66,67)
**R58**: N_2_(X, *v* = 15: 59) + O_2_(b) → N_2_O(X) + O(^1^D)	1.0 × 10^–20^	(66,67)
**R59**: N(^2^D) + NO(X) → N_2_O(X)	6 × 10^–17^	(65)
**R60**: N(^4^S) + NO_2_(X) → O(^3^P) + N_2_O(X)	1.1 × 10^–17^	(68,69)
**R61**: N_2_(A) + O_2_(X) → O(^3^P) + N_2_O(X)	7.8 × 10^–20^	(65)
**R62**: O(^1^D) + N_2_(X, *v* = 0) + N_2_(X) → N_2_O(X) + N_2_(X)	4.2 × 10^–46^*T*_g_^–0.88^	(70)
**R63**: O(^1^D) + N_2_(X, *v* = 0) + O_2_(X) → N_2_O(X) + O_2_(X)	4.2 × 10^–46^*T*_g_^–0.88^	(70)
**R64**: N(^2^D) + N_2_O(X) → N_2_(X, *v* = 0) + NO(X)	1.5 × 10^–17^ Exp[−570/*T*_g_]	(71)
**R65**: O(^1^D) + N_2_O(X) → 2NO(X)	7.2 × 10^–17^	(71)
**R66**: O(^1^D) + N_2_O(X) → N_2_(X, *v* = 0) + O_2_(X)	4.4 × 10^–17^	(71)
**R67**: N_2_(A) + N_2_O(X) → N_2_(X, *v* = 0) + N(^4^S) + NO(X)	10^–17^	(65)
**R68**: O^–^(^2^P) + N_2_(X, *v* = 0) → N_2_O(X) + e	10^–17^	(8)

a Rate coefficients are expressed in m^3^ s^–1^ and m^6^ s^–1^ for two-body and three-body reactions, respectively. Gas temperature, *T*_g_, is expressed in K. Mechanisms in **bold** are novel/corrected in comparison to previous studies.

**Table 5:** List of Reactions Describing Heavy Species Collisions Involving O(^1^S)^a^

process	rate coefficient (S.I.)	ref
**R69**: N_2_(A) + O(^3^P) → N_2_(X, *v* = 0) + O(^1^S)	2.1 × 10^–17^	(73,74)
**R70**: O(^1^S) + O(^3^P) → O(^1^D) + O(^1^D)	5.0 × 10^–17^	(74)
**R71**: O(^1^S) + N(^4^S) → O(^3^P) + N(^4^S)	1.3 × 10^–18^	(74)
**R72**: O(^1^S) + N_2_(X) → O(^3^P) + N2O(X)	1.0 × 10^–23^	(74)
**R73**: O(^1^S) + O_2_(a) → O(^1^D) + O_2_(b)	2.9 × 10^–17^	(74)
**R74**: O(^1^S) + O_2_(a) → 3O(^3^P)	3.2 × 10^–17^	(74)
**R75**: O(^1^S) + NO(X) → O(^3^P) + NO(X)	2.9 × 10^–16^	(74)
**R76**: O(^1^S) + NO(X) → O(^1^D) + NO(X)	5.1 × 10^–16^	(74)
**R77**: O(^1^S) + O_3_(X) → O_2_(X) + O_2_(X)	2.9 × 10^–16^	(74)
**R78**: O(^1^S) + O_3_(X) → O_2_(X) + O(^3^P) + O(^1^D)	2.9 × 10^–16^	(74)
**R79**: O(^1^S) + N_2_O(X) → O(^3^P) + N_2_O(X)	6.3 × 10^–18^	(74)
**R80**: O(^1^S) + N_2_O(X) → O(^1^D) + N_2_O(X)	3.1 × 10^–18^	(74)
**R81**^T^: O(^1^S) + NO_2_(X) → 2O(^3^P) + NO(X)	2.5 × 10^–16^	(76,77)
**R82** ^T^: O(^1^S) + N_2_O(X) → 2O(^3^P) + N_2_(X, *v* = 0)	2.5 × 10^–16^	(76,77)
**R83**^T^: O(^1^S) + N_2_O(X) → 2NO(X)	2.5 × 10^–16^	this work

a Rate coefficients are expressed in m^3^ s^–1^ and m^6^ s^–1^ for two-body and three-body reactions, respectively. Gas temperature, *T*_g_, is expressed in K. Mechanisms in **bold** are novel/corrected in comparison to previous studies. Reactions with superscript T (not included in the default chemistry module) are tested in this work with an upper limit rate coefficient that approximates the gas kinetic rate.

Tables 1–5 present the reactions associated with interactions between nitrogen and oxygen species, as considered in this paper. Within these tables, we have highlighted in **bold** the novel mechanisms in comparison with the previous studies. These updates incorporate several crucial changes associated with reactions involving electronically excited O(^1^D), O_2_(b), O(^1^S), and molecular NO(X) and N_2_O(X) species. It is important to note that earlier works (e.g., Pintassilgo et al.^39^) did not consider O(^1^D), hence the absence of O(^1^D) contributing to the formation of NO(X). The integration of the O(^1^D) in the chemistry of N_2_–O_2_ plasmas is now accounted for through reactions **R13** and **R43** in Tables 1 and 2, respectively. Additionally, we have also now considered NO(X) production via collisions involving O_2_(b) and N(^4^S), illustrated in **R44**, utilizing a rate coefficient proposed by Uddi et al.^64^ The rate coefficient of 2.5 × 10^–16^ m^3^ s^–1^ used for this mechanism approximates the gas kinetic rate and was initially proposed to align with the experimental peak of the NO(X) concentration in air/fuel nanosecond pulse discharges. Note that this reaction (shown in Table 2 with the symbol T) is not included in the default chemistry module. The impact of this rate coefficient on the results and, in particular, on NO(X) production is discussed later in the paper. Finally, it is worth noting that regarding the production of NO(X) through the recombination of NO_2_(X) with oxygen *O*(^3^P) (reaction **R56**), we have rectified the rate coefficient associated with this mechanism based on the values provided by Kossyi.^65^

Reactions associated with the production and destruction of N_2_O(X) are indicated in Table 4. Reactions **R57** and **R58** involve N_2_O(X) production through the recombination of vibrationally excited nitrogen with electronically excited O_2_, as proposed by Fraser et al.^66^ with a rate coefficient of 10^–20^ m^3^ s^–1^, consistently utilized in other works in literature.^67^ For reaction **R59**, describing N(^2^D) + NO(X) → N_2_O(X), the rate coefficient from Kossyi^65^ is applied. Note, however, that this mechanism should be used with caution due to energy conservation concerns arising from the involvement of two reactants in N_2_O(X) formation without the presence of a third body. Reactions **R60** and **R61** leading to the production of N_2_O(X), involving electronically excited N_2_(A) and NO_2_(X), are considered with constant rate coefficients retrieved from several references.^65,68,69^ The validity range of the rate coefficients associated with these mechanisms is limited to relatively low temperatures (below 400 K). For three-body mechanisms contributing to N_2_O(X) production (reactions **R62–R63** in Table 4), the rate coefficients used in this work were obtained from results involving absorption spectroscopy diagnostics in laser flash photolysis measurements.^70^ These rate coefficients were estimated with relative errors of approximately 30–40%. We do not anticipate significant contributions from three-body mechanisms leading to N_2_O(X) production under our low pressure conditions.

Reactions **R64–R67** pertain to the destruction of N_2_O(X) via collisions with electronically excited atoms/molecules. Their rate coefficients are retrieved from the chemical kinetics database of Herron and Green.^71^ These rate coefficients should also be used with caution due to their limited validity within a specific range of gas temperatures, typically below 400 K. The final mechanism, **R68**, associated with the recombination of negative oxygen ions with N_2_, leading to the formation of N_2_O(X), is considered with a rate coefficient provided by Fridman.^7^ We explored additional mechanisms contributing to the production or destruction of N_2_O(X) but decided to discard them from the default scheme proposed in this work, based on their limited observed contribution, as pointed out in the reference sources and confirmed through modeling in this study. An example is the reaction O_2_(b) + N_2_O(X) → NO(X) + NO_2_(X), studied by Dunlea et al.^72^ in the context of NO(X) formation in the atmosphere.

The inclusion of O(^1^S) in the chemistry module, previously overlooked in studies focusing on pure O_2_ discharges (see Dias et al.^42^), was motivated by the mechanism leading to its production via N_2_(A) colliding with atomic oxygen O(^3^P), as highlighted in **R69** in Table 5. This mechanism has been experimentally detected by Piper^73^ with a rate coefficient of 2.1 × 10^–17^ m^3^ s^–1^ (measured at room temperature). This rate coefficient was also reported in the work of Kossyi^65^ and was used in the modeling of microwave air plasmas.^74^ Interestingly, a very recent work related to the understanding of fast gas heating in CO_2_ discharges^75^ has also highlighted the significance of O(^1^S) species. We further accounted for the generation of the O(^1^S) from the ground state O(^3^P) through electron impact excitation, incorporating a cross-section within the Boltzmann module. For the mechanisms leading to the destruction of O(^1^S) (reactions **R70–R80**), we relied on the work of Tatarova et al.^74^ and references therein. However, caution should be exercised when considering these rate coefficients due to their limited range of validity, often associated with low/room temperature. Finally, we have also considered the reactivity between O(^1^S) with NO_2_(X) and N_2_O(X) leading to the production of the NO(X) and N_2_(X) species. These mechanisms (**R81–R83** in Table 5) have been suggested by Murakami et al.,^76^ utilizing estimations provided by the theoretical work of Vidmar et al.^77^ We have considered a rate of 2.5 × 10^–16^ m^3^ s^–1^ for these mechanisms, similar to what has been considered for the reaction between O_2_(b) with N(^4^S) leading to NO(X) production mentioned earlier. These reactions (shown in Table 5 with the symbol T) are also not included in the default chemistry module. It should be noted that Murakami et al.^76^ considered a rate coefficient of 1.0 × 10^–16^ m^3^ s^–1^ for these mechanisms. The overall influence of the mechanisms approximated with the gas kinetic rate on the results will be studied later in the paper.

### 3.3 Surface Description in N_2_–O_2_ Discharges

Beyond gas-phase chemistry in N_2_–O_2_ discharges, the loss and recombination of plasma species at the reactor walls, known as heterogeneous processes, play a crucial role in the overall plasma kinetics, as demonstrated in prior works^35,78,82,90^. In this context, the recombination of oxygen and nitrogen atoms is often characterized by the recombination probability γ_*i*_ (see eq 3), which depends on various experimental conditions such as wall material, cleanliness, roughness, and temperature. For a detailed illustration of the range of different recombination probability values associated with nitrogen and oxygen, readers can refer to Kutasi et al.^91^ In their study, Kutasi et al.^91^ also explored the potential for NO(X) production at the plasma reactor wall, incorporating a recombination probability dependent on the percentage of N atoms lost on the surface.

Motivated by the need to quantify the creation of NO(X) at the wall, we have coupled the volume chemistry with a mesoscopic formulation to describe the fractional coverage of adsorption sites associated with atomic oxygen, atomic nitrogen, and ground state molecules NO(X) and N_2_O(X). In this formulation, initially developed by Kim and Boudart,^92^ and recently reviewed in Marinov et al.,^93^ surface processes are treated in the same way as gas-volume chemical reactions, with creation and loss terms for the surface densities of adsorbed species and vacant adsorption sites written in a similar form as in eq 1. We have then considered several mechanisms of physisorption, thermal desorption from physisorption sites, chemisorption, Eley–Rideal (E-R) recombination (involving the arrival of a gas-phase species to an occupied site at the surface), occupation of chemisorption sites by diffusing physisorbed atoms, and Langmuir–Hinshelwood (L–H) recombination (involving the diffusion of an adsorbed species toward an occupied site at the surface). These mechanisms, listed in Table 6, are responsible for the conversion of several species from the gas-phase, namely, O(^3^P) and N(^4^S) and N_2_(X, *v* = 0), into O_2_(X), N_2_(X, *v* = 0), NO(X), and N_2_O(X).

**Table 6:** List of Elementary Reactions Describing Surface Kinetics for O_2_(X), N_2_(X), NO(X), and N_2_O(X) Formation^a^

process	surface parameters	ref
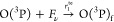	*k*_1_^0^ = 1, *E*_r_ = 0 kJ/mol	(56,95)
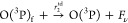	ν_d_ = 10^15^ s^–1^, *E*_d_ = 33.3 kJ/mol	(56,95)
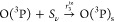	*k*_3_^0^ = 1, *E*_r_ = 0 kJ/mol	(56,95)
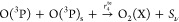	*k*_4_^0^ = 1, *E*_r_ = 25.5 kJ/mol	(56,95)
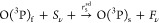	ν_D_ = 10^15^ s^–1^, *E*_D_ = 16.65 kJ/mol, *k*_5_^0^ = 1, *E*_r_ = 0 kJ/mol	(56,95)
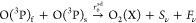	ν_D_ = 10^15^ s^–1^, *E*_D_ = 16.65 kJ/mol, *k*_6_^0^ = 1, *E*_r_ = 25.5 kJ/mol	(56,95)
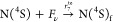	*k*_7_^0^ = 1, *E*_r_ = 0 kJ/mol	(56,95)
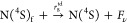	ν_d_ = 10^15^ s^–1^, *E*_d_ = 51 kJ/mol	(56,95)
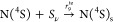	*k*_9_^0^ = 1, *E*_r_ = 0 kJ/mol	(56,95)
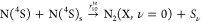	*k*_10_^0^ = 1, *E*_r_ = 20 kJ/mol	(56,95)
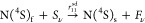	ν_D_ = 10^13^ s^–1^, *E*_D_ = 25.5 kJ/mol, *k*_11_^0^ = 1, *E*_r_ = 0 kJ/mol	(56,95)
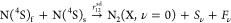	ν_D_ = 10^13^ s^–1^, *E*_D_ = 25.5 kJ/mol, *k*_12_^0^ = 1, *E*_r_ = 20 kJ/mol	(56,95)
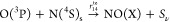	*k*_14_^0^ = 1, *E*_r_ = 25.5 kJ/mol	this work
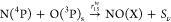	*k*_15_^0^ = 1, *E*_r_ = 20 kJ/mol	this work
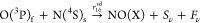	ν_D_ = 10^15^ s^–1^, *E*_D_ = 16.65 kJ/mol, *k*_16_^0^ = 1, *E*_r_ = 25.5 kJ/mol	this work
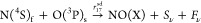	ν_D_ = 10^13^ s^–1^, *E*_D_ = 25.5 kJ/mol, *k*_17_^0^ = 1, *E*_r_ = 17.5–23 kJ/mol	this work
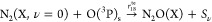	*k*_18_^0^ = 1, *E*_r_ = 25 kJ/mol	(98)

a Surface parameters are written according to eqs 5–7.

In Table 6, the species with subscripts f and s denote physisorbed and chemisorbed species, respectively, while *F*_*v*_ and *S*_*v*_ represent vacant physisorption sites and vacant chemisorption sites, respectively. The rate coefficients used to describe mechanisms are written (in dimensions of site ^–1^ s^–1^) according to the following three expressions associated with the thermal effusion toward the surface (*r*_*i*_^te^), thermal desorption from the surface (*r*_*i*_^td^), and surface diffusion (*r*_*i*_^sd^), according to
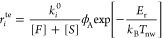
5
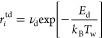
6
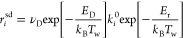
7where [*F*] and [*S*] are the surface densities of physisorption and chemisorption sites, respectively; *k*_*i*_^0^ are elementary sticking probabilities on physisorption/chemisorption sites; ϕ_A_ is the thermal flux of species A toward the surface (written as 1/4⟨*v*_A_⟩[A], via thermal speed ⟨*v*_A_⟩ and concentration [A]); *E*_r_ is the activation energy (*E*_r_ = 0 kJ/mol for adsorption mechanisms without recombination); *v*_d_ is the frequency factor for desorption; *E*_d_ is the energy barrier for desorption; ν_D_ is the characteristic diffusion frequency; and *E*_D_ is the energy barrier for diffusion. For the application of these expressions in this work, we used the following assumptions. We consider [*F*] = 10^20^ m^–2^ with a fraction of chemisorption sites of [*S*]/[*F*] ≃ 2 × 10^–3^. These values are supported by geometric considerations provided in Guerra et al.^10^ and experimental results of Kim and Boudart^92^ obtained in oxygen and nitrogen recombination in silica. Fractional values on the order of 10^–3^ were also proposed by Lefevre et al.^94^ in their study of nitrogen recombination in plasmas. Notably, as emphasized by these authors, the quantity of chemisorption sites may vary depending on the surface type, with Pyrex generally demonstrating a higher density of such sites. Caution is then advised when extrapolating or applying the previously mentioned values to a reactor surface that differs from silica.

To describe the recombination of nitrogen, we capitalize on the results of Gordiets et al.^95^ and we used *E*_r_ = 20 kJ/mol, *E*_d_ = 14 kJ/mol, *E*_D_ = 0.5*E*_d_, *v*_D_ = 10^15^ s^–1^, *v*_d_ = 10^13^ s^–1^, and elementary sticking probabilities *k*_*i*_^0^ = 1. According to these parameters, in pure *N*_2_ discharges, with a wall temperature of about 323 K, the recombination probability of nitrogen yields γ_*N*_ ≃ 3 × 10^–4^. To address the recombination of oxygen, we also capitalize on the results of Gordiets et al.^95^ and we used *E*_r_ = 25.5 kJ/mol, *E*_d_ = 33.3 kJ/mol, *E*_D_ = 0.5*E*_d_, *v*_D_ = *v*_d_ = 10^15^ s^–1^. According to these parameters, in pure O_2_ discharges, with a wall temperature of about 323 K, the recombination probability of nitrogen yields γ_O_ ≃ 2 × 10^–4^.

As compared to the previous assumptions, it is worth mentioning that studies found in literature,^93,96^ particularly focused on the description of oxygen recombination on Pyrex surfaces, have used slightly different input values associated with surface parameters, which include activation energies of *E*_d_ = 30 kJ/mol, recombination energies of *E*_r_ = 17 kJ/mol and frequency factors of *v*_D_ = 10^13^ s^–1^ and *v*_d_ = 10^15^ s^–1^. In another recent modeling study targeted at describing oxygen surface recombination on Pyrex surfaces, Viegas et al.^97^ achieved an excellent agreement with experimental data when using a fraction of chemisorption sites equal to 1.5 × 10^–2^ and [*F*] = 10^19^ m^–2^. A dedicated investigation aimed at reconciling surface parameters between pure oxygen plasmas and N_2_–O_2_ discharges, while understanding surface mechanisms tailored to different surfaces, falls beyond the scope of this study yet merits consideration in future research endeavors.

Regarding the formation of NO(X) at the reactor wall, the lack of fundamental data poses challenges for analysis and modeling. To overcome this limitation, the following assumptions were made. For E–R and L–H recombination leading to the formation of NO(X), we employed values based on pure oxygen and nitrogen recombination, setting *E*_r_ at 25.5 and 20 kJ/mol, for the arrival of oxygen and nitrogen atoms from the gas phase, respectively. However, in the case of the mechanism, N(^4^S)_f_ + O(^3^P)s → NO(X) + *S*_*v*_ + *F*_*v*_, we explored different energy recombination values *E*_r_ based on findings from Gordiets et al.^90^ While following the recommendations by these authors, we explored the impact of this L-H recombination leading to NO(X) formation within the 17.5–23 kJ/mol range (Table 6). Regarding N_2_O(X) formation at the wall through N_2_(X, *v* = 0) + O(^3^P)_s_ → N_2_O(X) + *S*_*v*_, we took into account the recombination energy *E*_r_ of 25 kJ/mol to achieve a recombination probability within the order of magnitude reported by Castillo et al.^98^ Note that according to Dean,^99^ the bond strength associated with N_2_O is given by D(ON–N) = 480.7 kJ/mol. This value leads to an upper limit for the activation energy of recombination (estimated via the exothermic step approximation given in Guerra et al.^100^) of 26 kJ/mol, which is very similar to the activation energy considered in this work. We further assumed that only ground state N_2_(X, *v* = 0) contributes to N_2_O(X) formation. Although not considered, this surface mechanism might also occur via highly excited vibrational levels of N_2_(X).

For electronically excited states, the wall diffusion is considered according to a surface loss probability, as indicated in Table 7. More specifically, for electronically excited states of N_2_, while following previous works,^35,39^ we assume a surface deactivation probability of 1. Note that the wall losses of atomic metastables N(^2^D) and N(^2^P) are also assumed to have a probability of 1. However, following the insights of Guerra et al.,^10^ we consider that part of the N(^2^D) and N(^2^P) losses (fraction of 10%) lead to the formation of N_2_(X, *v* = 0). For deactivation of vibrationally excited nitrogen, we assume a wall recombination probability γ_*v*_ = 1.1 × 10^–3^ following the measurements undertaken by Marinov et al.^101^ in Pyrex and fused silica surfaces. For wall recombination involving electronically excited states of O_2_, we considered the values provided in the reaction mechanism developed by Dias et al.,^42^ which considers recent measurements by Booth et al.^44^ We further considered the wall deactivation involving O(^1^S) leading to the production of O(^3^P) with a deactivation probability equal to 1.

**Table 7:** Reactions Describing the Transport of Neutral Species with Recombination Probabilities Taken Directly from the Literature^a^

process	γ_*i*_	ref
W1: N_2_(A) + wall → N_2_(X, *v* = 0) + wall	1	(35)
W2: N_2_(B) + wall → N_2_(X, *v* = 0) + wall	1	(35)
W3: N_2_(C) + wall → N_2_(X, *v* = 0) + wall	1	(35)
W4: N_2_(a) + wall → N_2_(X, *v* = 0) + wall	1	(35)
W5: N_2_(a′) + wall → N_2_(X, *v* = 0) + wall	1	(35)
W6: N_2_(^2^D) + wall → N(^4^S) + wall	0.9	(35)
W7: N_2_(^2^D) + wall → 0.5N_2_(X, *v* = 0) + wall	0.1	(35)
W8: N_2_(^2^P) + wall → N(^4^S) + wall	0.9	(35)
W9: N_2_(^2^P) + wall → 0.5N_2_(X, *v* = 0) + wall	0.1	(35)
W10: N_2_(X, *v* = 1: 59) + wall → N_2_(X, *v* = *v* – 1) + wall	1.1 × 10^–3^	(35,101)
W11: O_2_(a) + wall → O_2_(X) + wall	2.2 × 10^–4^	(42)
W12: O_2_(b) + wall → O_2_(X) + wall	0.135	(42)
W13: O_2_(Hz) + wall → O_2_(X) + wall	1	(42)
W14: O(^1^D) + wall → O(^3^P) + wall	1	(42)
W15: O_3_^*^ + wall → O_3_(X) + wall	0.1	(42)
**W16**: O(^1^S) + wall → O(^3^P) + wall	1	this work

a Mechanisms in **bold** are novel/corrected in comparison to previous studies.

### 3.4 Plasma Afterglow

For a better comparison between the modeling results and the measurement of NO(X) and N_2_O(X) species in the effluent of the plasma via mass spectrometry (see Figure 1), we considered the evolution of plasma species in the afterglow of the discharge. To describe the plasma afterglow, the present modeling simulations consider the initial values of densities and gas temperature derived under steady-state conditions as the starting point (depicted in Figure 1 at *z* = 0 cm) to solve the system of rate balance equations within the chemistry module. During the afterglow phase, the calculations are done according to several key assumptions: (i) the inelastic/superelastic electron rate coefficients, contingent on the electron energy distribution function, are assumed to be negligible due to their vanishing small role in the overall kinetics; (ii) the electron temperature is assumed to be equal to the gas temperature; and (iii) the quasi-neutrality is achieved by balancing the electron density, denoted as *n*_e_, with the densities of various ions. Note that the balance between electron density and ion densities is expressed as *n*_e_ = ∑_*j*_p__*N*_*j*_p__ – ∑_*j*_*n*__*N*_*j*_*n*__, where *j*_p_ and *j*_*n*_ represent single-charged positive ions and single-charged negative ions, respectively.

To incorporate spatial considerations throughout the afterglow phase, we further accounted for the translation of time into space, guided primarily by the gas flow rate. This approach is motivated by the need to align space-resolved measurements with the specific spatial location, where a catalyst may be positioned after the discharge ignition point. In Ma et al.,^27^ the catalyst was typically positioned approximately 10 cm away from the discharge coil. To address this point, we assume mass conservation while keeping constant the product between the mass density and the gas speed, i.e., ρ(*z*) · *q*_*v*_(*z*) = constant, where ρ(*z*) = (*m* · *p*)/(*k*_B_*T*_g_(*z*)). In this expression, *m* represents the gas mass, *p* is the gas pressure, *T*_g_(*z*) is the space-resolved gas temperature, *k*_B_ is the Boltzmann constant, and *q*_*v*_(*z*) is given by the product between the gas velocity *v*(*z*) and the cross-sectional area of tube *S*. This assumption implies considering the quantity *v*(*z*)/*T*(*z*) constant, resulting in the following time–space converting expression:
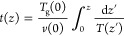
8where *T*_g_(0) and *v*(0) represent the gas temperature and the gas velocity at the beginning of the postdischarge. The gas velocity *v*(*z*) throughout the tube (expressed here in units of cm· s^–1^) is deduced from the pumping speed expression according to
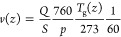
9where *Q* is the gas flow expressed in sccm units, *S* is the area expressed in cm^2^, *p* is the gas pressure expressed here in units of Torr, and *T*_g_(*z*) is the gas temperature expressed in units of Kelvin.

### 3.5 Working Conditions and Numeric Simulation

To conclude this section, we briefly overview the working conditions and considerations associated with the numerical simulation. Note that in addition to the gas pressure, reactor dimensions, initial mixture composition (taken from the experiment), and the volume/surface kinetic data (described in Sections 3.2 and 3.3), it is also required information related to the excitation frequency, the reduced electric field *E*/*N* (where *E* represents the electric field responsible for plasma maintenance and *N* represents the gas density), and the electron density *n*_*e*_ of the plasma. Concerning the excitation frequency, we follow the approach of Annusová et al.^49^ by assuming a DC field when solving the Boltzmann equation to obtain the electron energy distribution function. This approximation remains valid under the condition that the time modulation of the electron distribution function is minimal, resulting from an equilibrium between the angular frequency of the excitation field and the characteristic relaxation frequency for momentum transfer.^102^ However, it is important to note that, in contrast to the work of Annusová et al.,^49^ we consider a slightly higher pressure range (5 mbar vs 0.1 mbar), suggesting the potential influence of electron distribution function modulation at higher frequencies. A dedicated study targeted at understanding the effect of power modulation on electron energy distribution function goes beyond the scope of this work but should be considered in future studies. For the determination of *E*/*N*, several iteration loops are performed to ensure that the total electron creation rate compensates for its destruction rate, assuming a quasi-neutral discharge. Further calculations ensure (i) consistency of pressure (isobaric approximation) as defined by the experimental conditions, (ii) consistency between electron and chemistry modules by updating the steady-state gas mixture and gas temperature, and (iii) changes in the electron density to match the experimental power input and the calculated power. Concerning this third point, the calculated power is given by
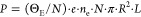
10where Θ_E_/*N* is the power density gained from the applied electric field and *e* is the electron charge. The workflow associated with these calculations can be found in previous works – see e.g., Sovelas et al.^41^ Relative tolerances associated with the determination of *E*/*N*, mixture composition, and electron density are all within a 10^–2^ factor. These tolerances, which are less conservative compared to previous works^42,50^ result from higher computational cost resulting from the inclusion of the complete set of N_2_ vibrations, along with the active thermal model and afterglow calculations.

## 4 Results

This section presents modeling and experimental results associated with a flowing N_2_–O_2_ discharge with varying oxygen content. While following the experimental input, the values of oxygen content used in the calculations range from zero, corresponding to a pure N_2_ environment, to 0.3, which is akin to an air-type mixture. It is important to mention that in our calculations, we have considered a default base chemistry model that takes into account all mechanisms specified in Tables 1–7. In this default base chemistry model, the recombination value of *E*_r_ = 17.5 kJ/mol is used for the N(^4^S)_f_ + O(^3^P)_s_ → NO(X) + *S*_*v*_ + *F*_*v*_ mechanism (for more details, refer to Section 3.3). Further considerations related to the influence of this particular reaction will be given later in the text. This section is divided into two parts. The first part outlines the results associated with the discharge, in which self-consistent calculations of the reduced electric field are performed for the various oxygen content conditions. The second part of this section involves the analysis of modeling results obtained for the postdischarge, concurrently comparing these outcomes with the available experimental data in terms of species produced.

### 4.1 Discharge

#### 4.1.1 EEDF and Species Densities

**Figure 4:**
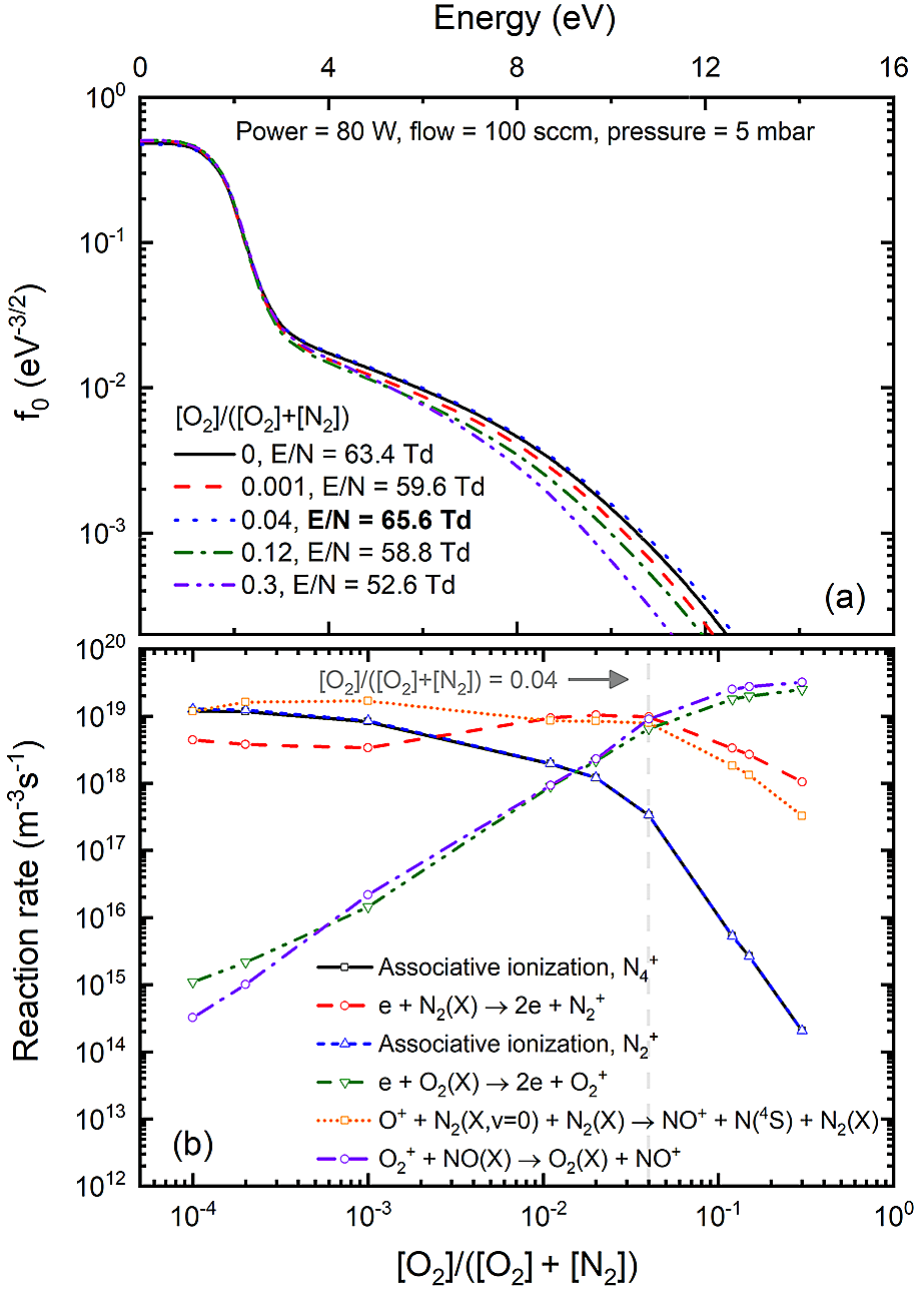
Electron energy distribution function for various oxygen content values (a) and reaction rates associated with various ionization mechanisms leading to the production of N_4_^+^(X), N_2_^+^(X), O_2_^+^(X), and NO^+^(X) (b). Open symbols indicated in (b) represent the oxygen content values for which the simulations were conducted. Vertical dashed line indicated in (b) represents the oxygen content values for which a maximum self-consistent reduced electric field is obtained. Results obtained for *p* = 5 mbar, total flow = 100 sccm, and *P* = 80 W.

We initiated our investigation by simulating the electron energy distribution function and the self-consistent reduced electric field across various oxygen content values (see Figure 4a). While previous research has explored the impact of oxygen addition on discharge characteristics,^39,81^ it is noteworthy that these earlier studies were primarily focused on either microwave discharge conditions or DC glow discharges modeled at constant currents. Similar results in terms of self-consistent *E*/*N* trends were obtained in this work and targeted at describing RF discharges at constant power. We observed a slight increase in *E*/*N* with the addition of 3–5% of O_2_. This increase compensates for the diminishing effect of Penning ionization resulting from collisions between metastable nitrogen molecules N_2_(A) and N_2_(a′), leading to the production of N_2_^+^(X) or N_4_^+^(X) ions (see Figure 4b). For higher O_2_ concentrations, ionization primarily results from electron impact involving N_2_(X), O_2_(X), and other mechanisms leading to NO^+^(X) formation, such as O_2_^+^(X) + NO(X) → O_2_(X) + NO^+^(X). For increasing oxygen concentrations, NO^+^(X) becomes the predominant ion in the discharge. Indeed, with an oxygen content of 0.3, the density of NO^+^(X) is approximately 10^16^ m^–3^ – roughly 2 orders of magnitude higher than that of O_2_^+^(X). Key mechanisms driving the production of NO^+^(X) are illustrated in Figure 4b.

Finally, regarding the results shown in Figure 4, two remarks warrant attention. First, the calculated *E*/*N* values are significantly influenced by associative ionization leading to the formation of N_2_^+^(X) or N_4_^+^(X) ions, which, in turn, relies on the concentration of N_2_(A). As previously mentioned, in the production of N_2_(A), we assume single energy-loss processes involving solely the ground state level N_2_(X, *v* = 0). Future improvements dedicated to the development of a reaction mechanism for N_2_ discharges should validate the presented *E*/*N* values and assess their dependence on verified N_2_(A) concentrations, which are currently unavailable for the system studied in this work. Second, concerning the electron energy distribution function, note that with the increasing oxygen content we observe a depletion of the distribution tail, corresponding to the reduction in the reduced electric field. This point is supported by the lower ionization threshold of oxygen compared to nitrogen, causing a decrease in the reduced electric field when oxygen is introduced into the discharge, and associative ionization is no longer significant. The decline in the electron energy distribution function around 2.5 eV can be attributed to the pronounced peak in the N_2_ total vibrational excitation cross-section at this energy, acting as a vibrational barrier that restricts electrons from reaching higher energies.

**Figure 5:**
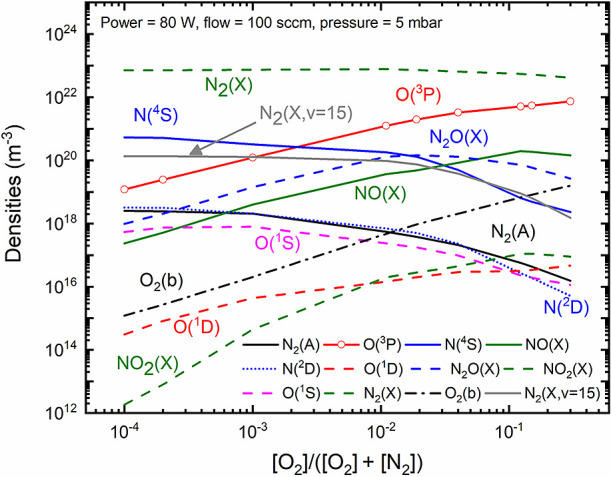
Concentrations of the main plasma species obtained in N_2_–O_2_ discharges as a function of the oxygen content. Empty symbols represent the oxygen content values for which the simulations were conducted. Results obtained for *p* = 5 mbar, total flow = 100 sccm and *P* = 80 W.

In Figure 5, we present the modeling results associated with the primary neutral species generated in the discharge. For conditions with a high nitrogen content, there is a significant production of atomic nitrogen N(^4^S); however, the introduction of oxygen results in the quenching of N(^4^S) and a linear increase in O(^3^P). NO(X) production also exhibits a linear increase until an oxygen content of approximately 0.1. In line with modeling results reported in the literature,^78,81,103^ a further increase in oxygen content (not depicted in this work) leads to a reduction in NO(X) production. Note that typical maximum values of NO(X) production in plasmas are often reported for oxygen content values ranging from 0.2 to 0.4.^103^ Experimental data^22^ associated with microwave discharges reported a maximum NO(X) production (measured downstream from the plasma source with FTIR) at an oxygen content of about 50%. It is also worth mentioning that for the conditions shown in Figure 5, the gas temperatures (calculated through eq 4) revealed an increase with the amount of oxygen content, ranging from roughly 400 to 580 K. Similar observations were also reported by Pintassilgo et al.,^37^ and they are related to an increase of the contribution associated with collisions between oxygen atoms and vibrationally excited N_2_(X).

In terms of electronically excited states, it is relevant to notice that nitrogen-related species (such as N_2_(A) and N(^2^D)) have higher density under conditions approaching pure nitrogen content, while the density of O(^1^D) increases with rising oxygen content. Interestingly, the concentration associated with O(^1^S) reaches its maximum density under conditions where N_2_ is predominant, owing to the transfer between N_2_(A) and O(^3^P) via N_2_(A) + O(^3^P) → N_2_(X, *v* = 0) + O(^1^S) (refer to **R69** in Table 4). The results from the modeling conducted by Murakami et al.,^76^ investigating atmospheric pressure helium–oxygen plasmas with humid air impurities, showed concentrations of O(^1^S) comparable to those of O(^1^D). This observation aligns with our findings, particularly when oxygen content values approach air-like mixtures. Finally, it is worth noting that the concentration of N_2_O(X) begins to decrease as oxygen content values increase. This decline is primarily attributed to the elevated presence of O(^1^D), which effectively quenches N_2_O(X) through mechanisms **R65** and **R66**, as outlined in Table 4. The contribution of these mechanisms to the destruction of N_2_O(X) increases by approximately 50% under high oxygen content conditions compared to under low oxygen content conditions. Furthermore, it is noteworthy to observe the prevalence of vibrationally excited N_2_(X), across the entire range of oxygen content, with concentrations exceeding 10^18^ m^–3^, as exemplified for the N_2_(X, *v* = 15) state. Note that these vibrationally excited states actively participate in volume reactions, leading to the destruction of atomic oxygen, O(^3^P), and the production of NO(X), as illustrated and discussed below.

#### 4.1.2 Creation/Loss Mechanisms

**Figure 6:**
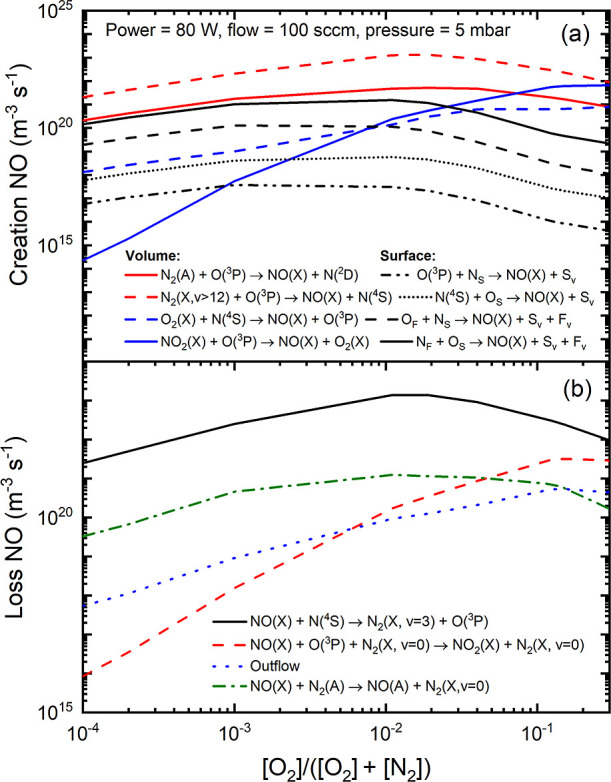
Main mechanisms leading to the creation (a) and destruction (b) of NO(X) molecules in the plasma. Mechanisms illustrated in (a) with black (color) curves are related to surface (volume) mechanisms. Results are obtained for *p* = 5 mbar, total flow = 100 sccm, and *P* = 80 W. In (b), the outflow represents the loss of NO(X) molecules through the flow of species out of the reactor.

Figure 6 provides a visual of the primary processes occurring in the volume and at the surface, shedding light on both the generation and destruction of NO(X) in the plasma. These contributions obtained within the discharge phase are associated with the concentrations highlighted in the preceding figure. In Figure 6, we observe that volume mechanisms leading to the creation of NO(X) dominate throughout the total range of oxygen content. In particular, it is worth noting the domination of the mechanisms leading to NO(X) production via collisions of atomic oxygen O(^3^P) with either N_2_(A) or vibrationally excited N_2_ via the Zeldovich mechanism (**R14**). The small maximum observed for the mechanism N_2_(X, *v* = 13:59) + O(^3^P) → NO(X) + O(^3^P) comes from the balance between the decrease of vibrational excitation and increase of atomic oxygen production, while the content of molecular oxygen gas fraction increases. Notice also that these mechanisms are also balanced by the destruction of NO(X) at the volume of the plasma via collisions with atomic nitrogen N(^4^S) (see Figure 6b). Interestingly, the decrease of N(^4^S) with the increase of oxygen content is not strong enough to explain the sharp increase of experimentally observed NO(X) (see, e.g., Figure 2 in Section 3.1). Finally, it is also worth noticing that for high oxygen content, we may also expect a contribution of mechanisms involving the density redistribution among NO_*x*_ molecules, which in Figure 6 is represented by the mechanism NO_2_(X) + O(^3^P) → NO(X) + O_2_(X).

**Figure 7:**
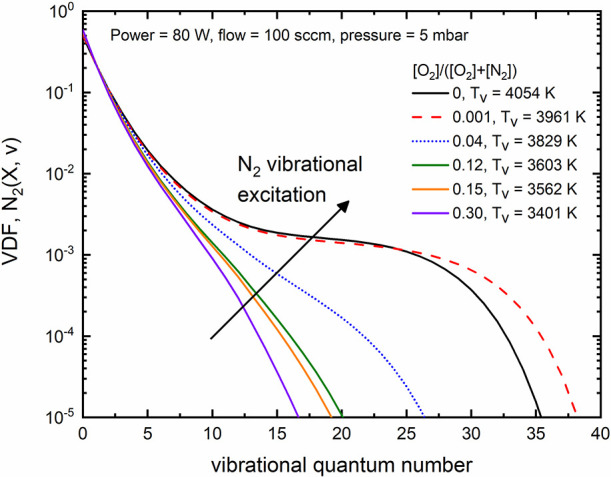
Vibrational distribution of N_2_(X, *v*) (normalized to the total N_2_(X) concentration) for different oxygen content values. Results obtained for *p* = 5 mbar, total flow = 100 sccm, and *P* = 80 W. Vibrational temperature (*T*_*v*_) values indicated are determined based on the very first excited vibrational level assuming a Boltzmann distribution.

Given the significance of vibrationally excited N_2_ in contributing to the production of NO(X), it is also valuable to analyze the vibrational distribution function associated with N_2_ obtained in this work. Figure 7 illustrates the evolution of the vibrational distribution linked to N_2_ for various oxygen content values. For discharges sustained on either pure N_2_ or with high nitrogen content, we observe a distinct plateau in the vibrational distribution between levels *v* = 15 and *v* = 30. This plateau is followed by a decline toward the tail of the distribution. These distribution characteristics arise from a combination of electron–vibration (e–V) and vibration–vibration (V–V) exchanges dominating at lower vibrational levels, resonant V–V exchanges at intermediate levels, and vibration–translation (V–T) exchanges involving N_2_ molecules and N atoms at higher levels. Similar distributions were observed in Guerra et al.^81^ Increasing the oxygen content leads to the quenching of high vibrational levels, resulting in distributions resembling a Maxwellian-like shape.

#### 4.1.3 Effect of the Power

**Figure 8:**
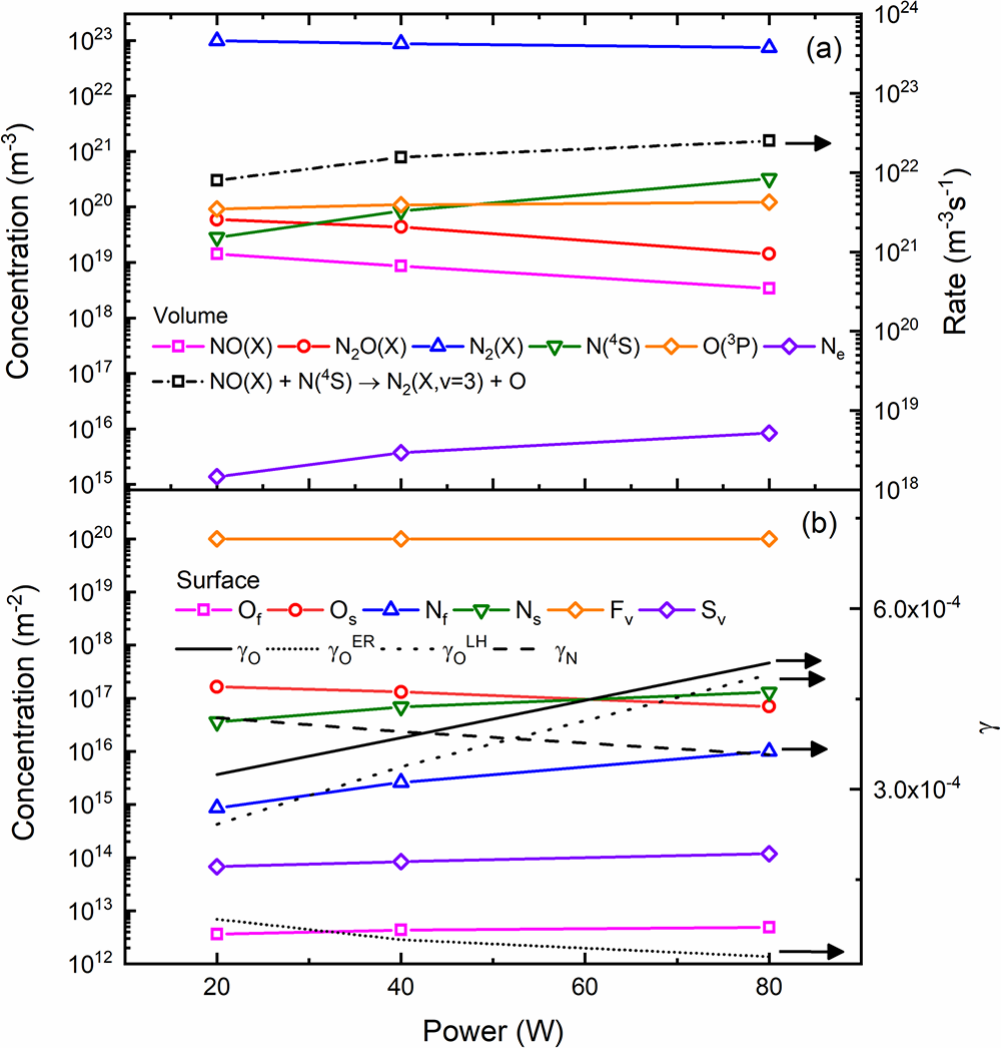
Left: Plasma species of relevance to the creation of NO(X), as a function of the input power, in volume (a) and at the surface (b). Right: Reaction rate relative to the production of NO via N(^4^S) collisions (a) and recombination probabilities associated with the formation of oxygen and nitrogen (b). Results obtained for *p* = 5 mbar, total flow = 100 sccm, and [O_2_]/([O_2_] + [N_2_]) = 0.001.

The impact of power variations on plasma species produced is examined in this section, considering different power values as inputs for estimating the electron density (see eq 10 in Section 3.5). This investigation is motivated by the necessity to explore how varying power levels may affect NO(X) production, while also bringing insights into the relationship between chemical reactions and surface kinetics. While conducting a parametric study as a function of the power input, we compared the results at *P* = 80 W with modeling outcomes at *P* = 40 and 20 W (see Figure 8), representing 50 and 25% of the default power, respectively. Overall, we observe a natural increase in electron density with rising power. However, concerning NO(X) production, higher power does not necessarily translate to higher NO(X) production. The increasing dissociation of N_2_(X) resulted in a higher concentration of atomic nitrogen N(^4^S), leading to the loss of NO(X) due to the increasing contribution of the process NO(X) + N(^4^S) → N_2_(X, *v* = 3) + O(^3^P) (also shown in Figure 8). Due to the minimal variation observed (particularly in terms of NO(X) production) across the power range studied, we opted to maintain a constant power level of 80 W for the remainder of this study. To complement the volume-related findings, Figure 8b also includes the concentration of species adsorbed at the surface wall (expressed in units of m^–2^). Additionally, Figure 8b presents the calculated recombination probabilities associated with the oxygen and nitrogen production. The recombination probabilities of oxygen γ_*O*_ and nitrogen γ_*N*_ are calculated according to
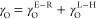
11where
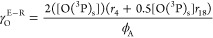
12
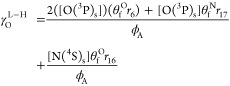
13and
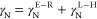
14where
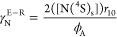
15
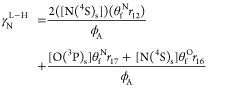
16We have included here the various contributions involving E–R and L–H recombination mechanisms with the various rates *r*_*i*_ considered in Table 6. The terms θ_f_^N^ and θ_f_^O^ represent the fractional coverage of the physisorption site associated with nitrogen and oxygen, respectively. Unlike the description presented in Guerra^56^ (focused on either pure O_2_ or pure N_2_ situations), E–R recombination mechanisms considered in this work can also lead to the loss of adsorbed oxygen via recombination with the N_2_(X, *v* = 0) forming N_2_O. At the same time, L–H recombination mechanisms considered for the calculation of recombination probabilities take into account not only the production of N_2_(X) and O_2_(X) species but also the production of NO(X) (see Table 6).

The impact of these surface mechanisms becomes apparent in Figure 8b, where some variations in probability values are evident, corresponding to changes in dissociation fractions obtained at different power levels. These small variations mirror the subtle changes in atomic oxygen and atomic nitrogen (and also NO(X) and N_2_O(X)) concentrations observed across the range of power values studied. The recombination probabilities obtained in our study support the findings of Guerra^56^ and Gordiets et al.,^90^ confirming the order of magnitude for recombination probabilities (in both O_2_(X) and N_2_(X)) of about 10^–4^ obtained for typical wall temperatures of 323 K. It is also worth noticing that for the range of powers studied, the ratio between O(^3^P) and N(^4^S) concentrations ([O(^3^P]/[N(^4^S)]) increases a factor of 10 (roughly from 0.5 to 4) when the power decreases from 80 to 20 W. Following the results of Gordiets et al.^78^ and also verified in this work, this increase of [O(^3^P]/[N(^4^S)] leads to a linear increase of the O_2_(X) recombination probability. The strength of this increase is related not only to surface parameters leading to the formation O_2_(X) or N_2_(X) but also to surface parameters leading to the production of NO(X) via, for example, N(^4^S)_f_ + O(^3^P)_s_ → NO(X) + *S*_*v*_ + *F*_*v*_ (reaction **17** in Table 6).

In conclusion, these results support our confidence in describing surface mechanisms, which are now integrated throughout the postdischarge phase, as discussed in the subsequent section. It is worth noting that some studies^33^ have suggested a potential reduction in recombination probabilities when transitioning from the discharge stage to the postdischarge region. This reduction could justify the adjustments discussed in the following sections. However, to the best of our knowledge, no experimental study has conclusively confirmed such a significant decrease in surface recombination probabilities during the afterglow phase.

### 4.2 Postdischarge

#### 4.2.1 NO(X) and N_2_O(X) Formation

Having calculated the species concentration under steady-state conditions in the discharge, we proceeded with the analysis of the postdischarge, taking into account the assumptions described in Section 3.4. It is important to highlight that in the postdischarge, the gas temperature relaxes to a wall temperature set at 323 K, as imposed by eq 4 and as described in Section 3.1. By utilizing eq 8, we can now assess the concentration of different species at various positions (referred to here as *z*; see Figure 1) within the reactor. The position *z* = 0 cm is associated with the results obtained in the discharge, serving as initial conditions for evaluating the species’ concentrations in the postdischarge.

**Figure 9:**
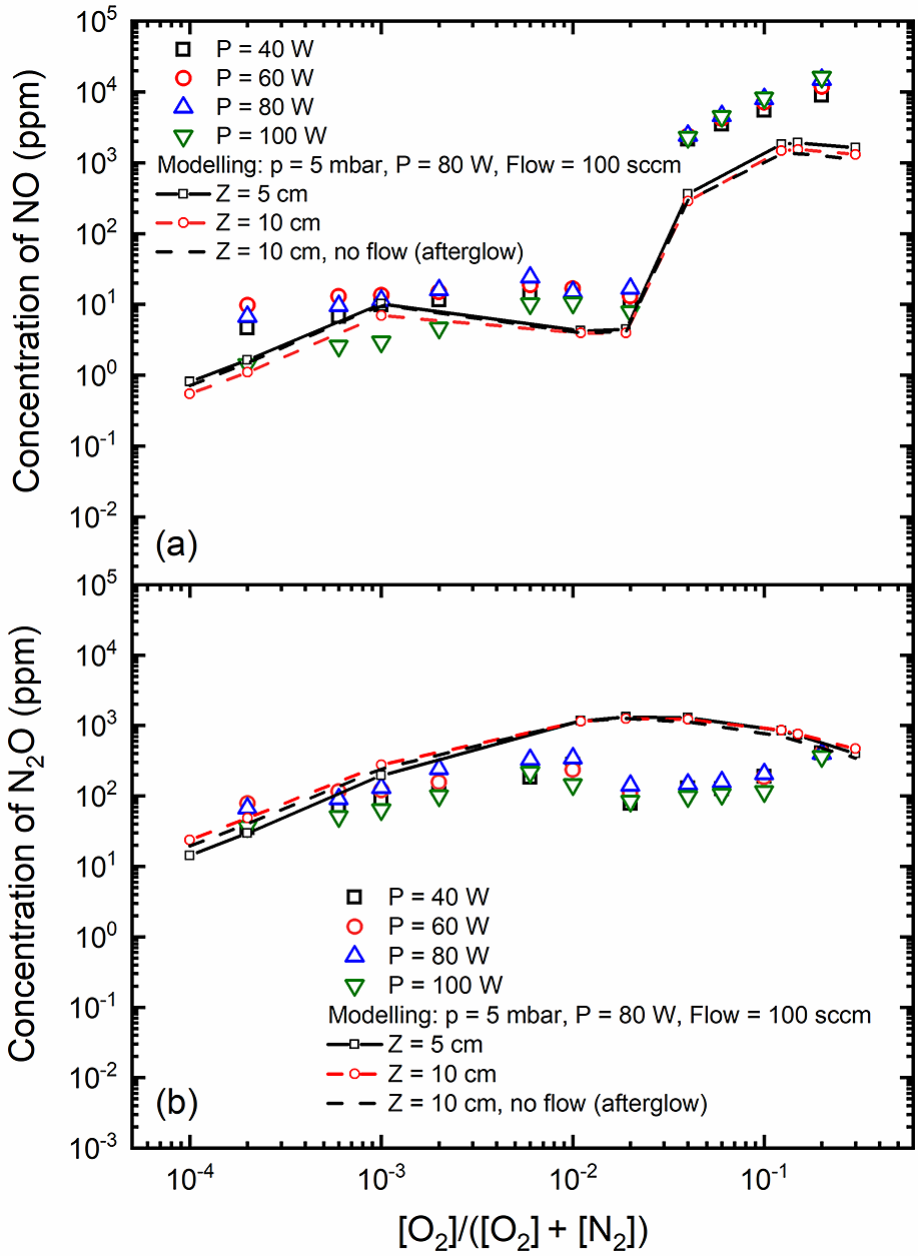
Comparison of modeling results and experimental data for the concentration of NO(X) (a) and N_2_O(X) (b), presented in units of parts per million (ppm). Experimental data (measured via mass spectrometry in the afterglow of the plasma) were obtained for different power values, ranging from 40 to 100 W. Experimental error is of the same order of magnitude (about 5%) as the results previously shown in Figure 3. Modeling results were obtained for two different axial *z* positions (solid black, *z* = 5 cm; dashed red, *z* = 10 cm) of the afterglow. Dashed black lines are modeling results obtained at *z* = 10 cm in the absence of outflow in the afterglow.

In Figure 9, we depict the experimental evolution of NO(X) and N_2_O(X) as a function of oxygen content. Experimental data were collected for various power values through mass spectrometry, while the modeling results were obtained for a single power value (*P* = 80 W) at two different positions, *z* = 5 cm and *z* = 10 cm. These positions correspond to approximately 100 and 200 ms into the postdischarge, respectively. The location at *z* = 10 cm also falls within the permissible range for placing the catalyst within the reactor. Regarding the experimental outcomes obtained at lower oxygen content, it is noteworthy that a marginal reduction in NO(X) production occurs with an increase in power input. This observation reinforces the findings of the parametric study conducted in the preceding section, confirming the adverse impact of power on NO(X) production. Notably, it is also worth pointing out that, irrespective of the power used to initiate the discharge, a substantial increase in NO(X) is experimentally observed around an oxygen content of approximately [O_2_]/([O_2_] + [N_2_]) = 0.04. This observation aligns with results reported by Ma et al.,^27^ as briefly described in Section 3.1. Figure 9 vividly illustrates the remarkable agreement between the modeling outcomes and experimental data regarding both the NO(X) and N_2_O production. The model adeptly replicates the distinctive knee-like shape evident at oxygen content values of [O_2_]/([O_2_] + [N_2_]) = 0.04. Notably, there is an underestimation of the NO(X) concentration at higher oxygen content, possibly attributable to a deficiency in mechanisms responsible for NO(X) production, as elaborated below. It is also noteworthy that the model captures a subtle experimental decrease in N_2_O(X) at elevated oxygen content, although to a modest extent. Lastly, in Figure 9, we have incorporated supplementary results illustrating the influence of the outflow term. Specifically, we excluded the outflow contribution in the postdischarge, resulting in a slight decrease in NO(X) and an increase in N_2_O(X) production.

**Figure 10:**
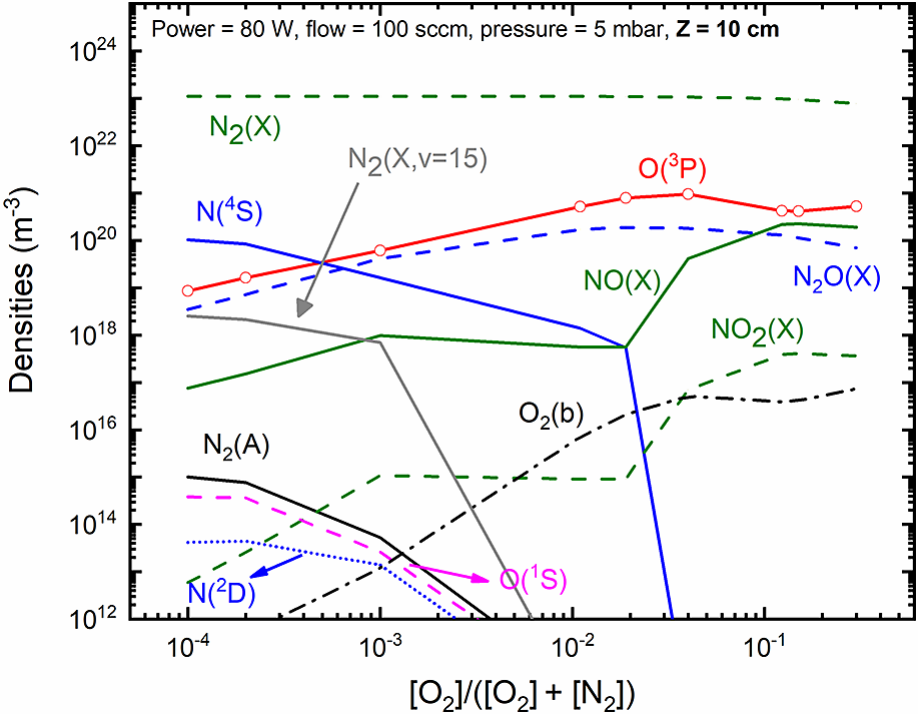
Concentrations of the main plasma species obtained in N_2_–O_2_ discharges as a function of the oxygen content in the afterglow (*z* = 10 cm). Results were obtained for *p* = 5 mbar, total flow = 100 sccm, and *P* = 80 W. Open symbols indicate the oxygen content values for which the simulations were conducted.

To enhance the comprehensiveness of our analysis, we have analyzed the concentrations of various neutral species as a function of oxygen content in the afterglow, see Figure 10 at a position *z* = 10 cm. A notable observation is the pronounced decrease in atomic nitrogen density with increasing oxygen content. This decline is particularly evident following the increase in NO(X) and NO_2_(X) concentrations. A strong reduction in the population of electronically excited molecules and atoms is also evident when contrasted with the discharge results previously showcased in Figure 5. It is worth mentioning that the concentration of O(^1^D) is omitted from this figure due to its low concentration, falling below 10^12^ m^–3^. For a low oxygen content, it is notable that vibrationally excited N_2_ can reach concentrations exceeding 10^18^ m^–3^, thereby playing a significant role in the production of NO(X) or N(^4^S) throughout the afterglow. This point is further elaborated below.

#### 4.2.2 Creation/Loss Mechanisms in the Postdischarge

**Figure 11:**
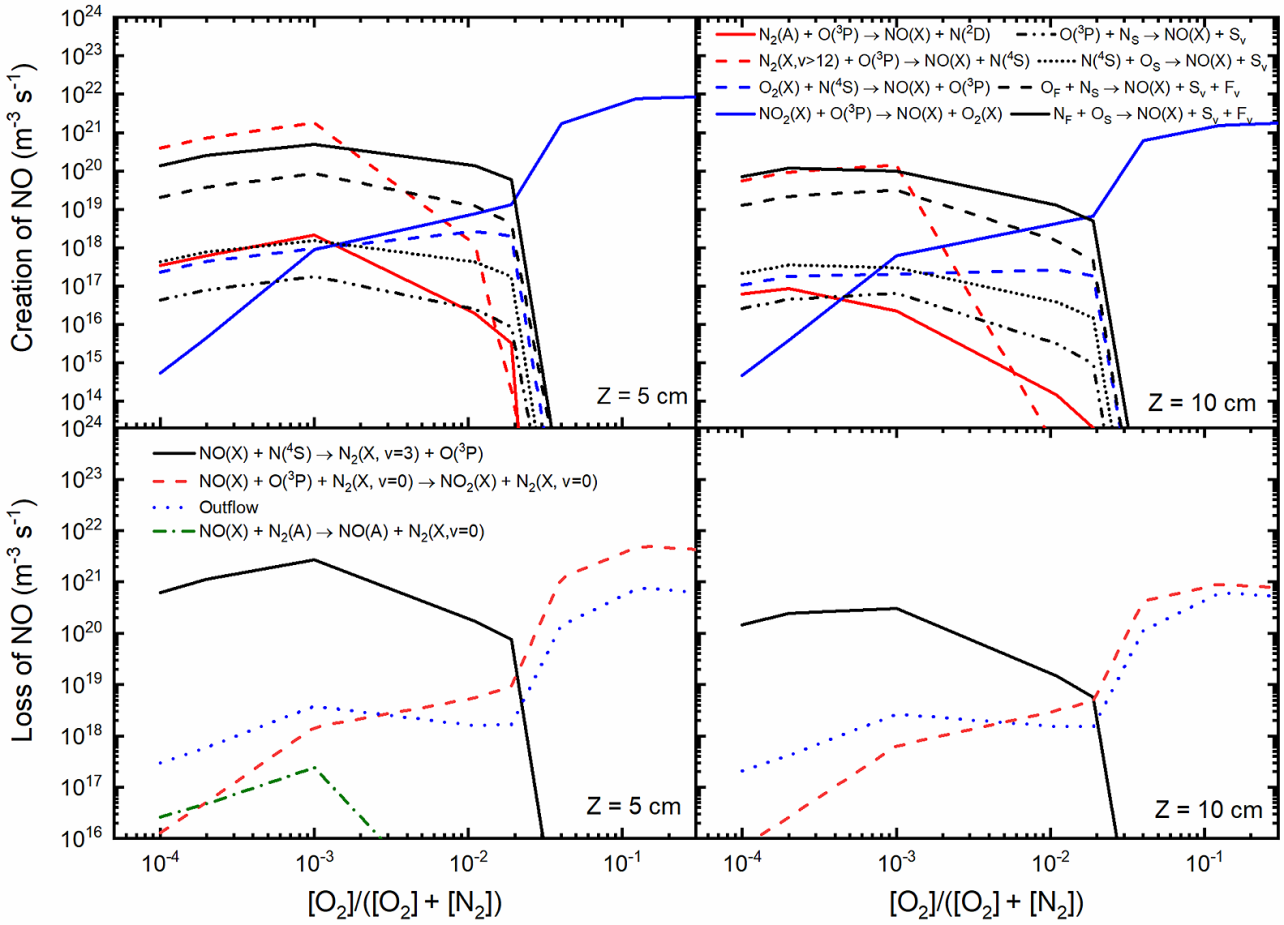
Main mechanisms leading to the creation and destruction of NO(X) molecules in the afterglow of plasma at two different positions, *z* = 5 and 10 cm. For NO(X) production, the mechanisms illustrated with black (color) curves are related to surface (volume) mechanisms. Results are obtained for *p* = 5 mbar, total flow = 100 sccm, and *P* = 80 W. Outflow represents the loss of NO(X) molecules through the flow of species out of the reactor.

To comprehend the evolution of NO(X) in the postdischarge, a closer examination of the primary mechanisms governing the production and loss of NO(X) at positions *z* = 5 cm and *z* = 10 cm is essential. In Figure 11, we illustrate the reaction rates for various mechanisms associated with the creation and loss of NO(X). Notably, unlike the predominance of volume mechanisms presented in the discharge, we now observe the potential dominance of surface mechanisms contributing to NO(X) production. A noteworthy observation is the significant contribution of N(^4^S)_f_ + O(^3^P)_s_ → NO(X) + *S*_*v*_ + *F*_*v*_ to the production of NO(X) spatially at *z* = 10 cm, particularly for oxygen content ranging from approximately 10^–3^ to 10^–2^. It is important to remember that a recombination energy of *E*_r_ of 17.5 kJ/mol is assumed for this reaction. Given the uncertainty surrounding this recombination energy, we will later explore the influence of this mechanism on the results.

In terms of NO(X) production mechanisms, it is worth noting the decreasing contribution of N_2_(X, *v* = 13:59) + O(^3^P) → NO(X) + O(^3^P) for increasing oxygen content as a result of the strong vibrational quenching throughout the afterglow. The decreasing contribution of this mechanism with the distance, from *z* = 5 cm to *z* = 10 cm, is also visible in Figure 11. Concerning the destruction of NO(X), it is important to notice the sharp decrease of the NO(X) + N(^4^S) → N_2_(X, *v* = 3) + O(^3^P) mechanism. This decrease aligns well with the pronounced increase in NO(X) concentration around 0.04. Indeed, it can then be inferred that the experimental increase in NO(X) concentration at low oxygen content is closely linked to the decrease in atomic nitrogen atoms density during the afterglow. This observation further supports the assumption that the mechanisms influencing NO(X) production, with and without catalyst, as depicted in Figure 3, may involve an important contribution of atomic nitrogen atoms adsorbed at the surface. For high oxygen content and similar to what was observed in the discharge, we may also expect a contribution of mechanisms for NO(X) production involving the density redistribution among NO_2_(X) molecules.

#### 4.2.3 Atomic Nitrogen Influence

**Figure 12:**
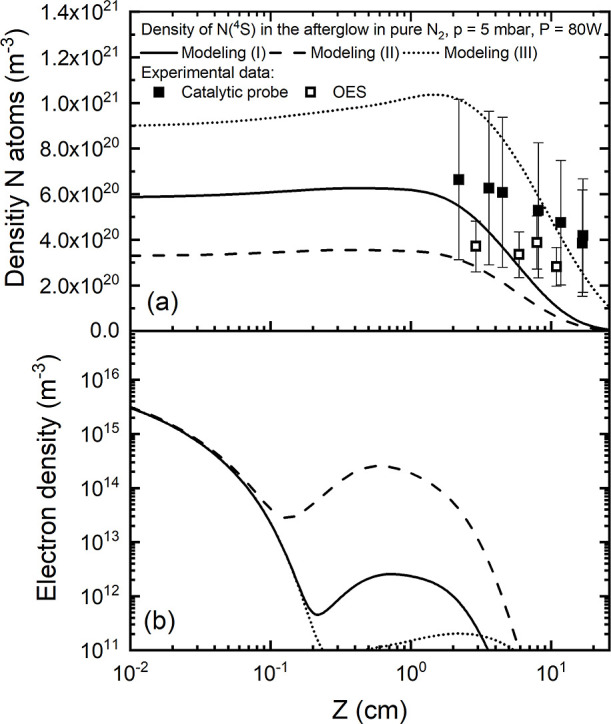
Calculated atomic nitrogen density (a) and electron density (b) as a function of distance in the postdischarge. Experimental measurements for atomic nitrogen were taken as close to the plasma as possible, with the initial experimental point assumed to mark the initiation of the afterglow. The various lines are obtained for different modeling assumptions associated with the rate coefficient associated with the dissociation of N_2_ via N_2_(X, *v* = 13:59) + O(^3^P) → NO(X) + O(^3^P) and recombination energy of for N(^4^S) + N(^4^S)_s_ → N_2_(X, *v* = 0) + *S*_*v*_.

While recognizing the crucial role of atomic nitrogen atoms in NO(X) production/destruction, we compared modeling results against experimental data associated with N(^4^S) production, detected along the afterglow, in pure N_2_ conditions, as depicted in Figure 12. The experimental results, shown in this figure, were obtained by catalytic probe measurements. The probe consisted of two thermocouples mounted through an adjustable feedthrough, giving a range of motion of approximately 18 cm. One of the thermocouples was coated with a thin catalytic layer, promoting the exothermic recombination of radicals, while the other served as a reference, obtaining information on plasma interactions and heat fluxes. The density of radicals was then inferred from temperature differences and knowledge of recombination rates, following the methodology outlined by Mozetić et al.^104^ and Qerimi et al.^105^ These probe-based experimental results were further compared against data obtained from OES, specifically utilizing first positive system measurements, alongside intensity calibrations and the approximations detailed in Peeters et al.^106^

Given the uncertainty regarding the plasma boundary and while considering that these measurements were done as close to the plasma as possible, we assume that the initial experimental data mark the beginning of the afterglow. Furthermore, and in alignment with insights from Guerra et al.,^35^ particularly concerning uncertainties in N_2_ dissociation, we have investigated the impact of the rate coefficient for N_2_(A) + N_2_(X, *v* = 15:19) → N_2_(X, *v* = 0) + 2N(^4^S). In the default scenario (Modeling I), represented in Figure 12, we utilized a rate coefficient of 4.5 × 10^–17^ exp[−1765/*T*_g_] m^3^ s^–1^, while in Modeling II, we used a constant rate coefficient of 5.0 × 10^–19^ m^3^ s^–1^. These rate coefficients were used in previous works dedicated to the study of N_2_ discharges – see Guerra et al.^35^ In Modeling III, we maintained the same gas–temperature-dependent rate coefficient as in the initial default scenario, while increasing the recombination energy associated with the N(^4^S) + N(^4^S)_s_ → N_2_(X, *v* = 0) + *S*_*v*_ mechanism. Originally considered with *E*_r_ = 20 kJ/mol (refer to Table 6), we adjusted here the recombination energy of this mechanism to *E*_r_ = 23 kJ/mol. This third adjustment stems from uncertainties in recombination energies for N_2_ formation, as discussed in,^56^ where a range of *E*_r_ between 14 and 20 kJ/mol was explored. The choice of the rate coefficient for this mechanism can yield significant differences in the atomic nitrogen production. Consequently, the evolution of atomic nitrogen throughout the afterglow strongly influences electron density (calculated here via the sum of ion densities) (see Figure 12b). It is worth noticing that preliminary experimental estimations of the electron density (not shown) acquired through Langmuir double probe measurements yield values within the 10^14^ m^–3^ of magnitude, which falls within the range of values obtained through our model in the earlier afterglow. Similar to the measurements of nitrogen atom density, the detection of electron density was also carried out as close to the end of the plasma column as feasible, with data points collected between 1 and 20 cm past the plasma termination point. Finally, it is worth pointing out that with these three modeling situations, we obtain a fraction of N(^4^S) atoms that fall within the range between 0.46 and 1.27% of the total N_2_(X) concentration. For comparison, experimental and modeling results from Volynets et al.^107^ in DC glow discharges, conducted at similar pressure conditions (5 Torr) and electron densities (corresponding to currents of *I* = 50–90 mA), reveal N(^4^S) fractions ranging between 0.66 and 2.7%. Overall, these results support the validity of the model. However, it is important to emphasize that dedicated investigations aimed at defining the reaction mechanisms in N_2_ and N_2_–O_2_ discharges, along with conducting a systematic comparison with the existing literature, are still necessary.

#### 4.2.4 Model Improvement

**Figure 13:**
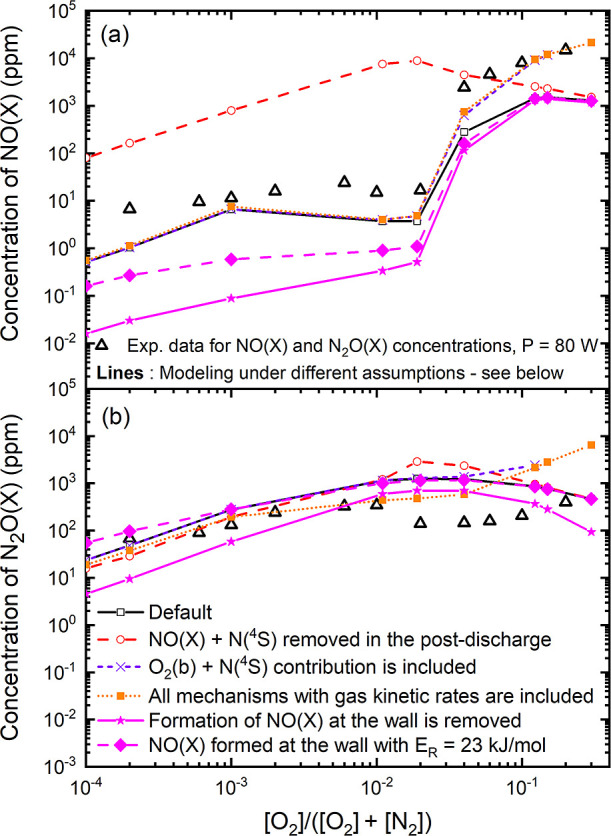
Comparison between measured and calculated concentration of NO(X) (a) and N_2_O(X) (b) molecules at *Z* = 10 cm. Experimental data refer to the condition with a power *P* = 80 W. Experimental error is of the same order of magnitude (about 5%) as the results previously shown in Figure 3. Default modeling condition (black line) is compared against several simulation assumptions in which different reactions and rate coefficients are taken/removed from the kinetic scheme – see text for more details.

In this section, we aim to elucidate the impact of various mechanisms governing the production of NO(X) and N_2_O(X) during the afterglow. Figure 13 provides a comparison between experimental data for NO(X) and N_2_O(X) at *P* = 80 W and modeling results undertaken with different assumptions. The default condition (represented by the solid black line) aligns with the parameters outlined in Section 4.1. All other assumptions are built on top of this default condition. We initially analyze the results in terms of NO(X) and N_2_O(X) production without including the recombination mechanism NO(X) + N(^4^S) → N_2_(X, *v* = 3) + O(^3^P) (reaction **R34** in Table 2) in the afterglow. Omitting this mechanism results in a significant overestimation of NO(X) production, particularly evident at low oxygen content, and eliminates the knee-like shape observed in the experimental data (see points in Figure 13). Intriguingly, at high oxygen content, the NO(X) production under this assumption converges toward the default case. At high oxygen content, the significance of this recombination mechanism decreases, primarily due to the diminishing importance of vibrationally excited N_2_ (see Figure 7) particularly noticeable during the afterglow. Note that N_2_O(X) also becomes more overestimated (compared with the default situation) when neglecting this recombination.

Subsequently, we explored the effect of O_2_(b) + N(^4^S) → NO(X) + O(^3^P) (reaction **R44** in Table 2). The inclusion of this mechanism leads to an improvement in terms of NO(X) produced for high oxygen content values. This mechanism, suggested by Uddi et al.^64^ to explain NO(X) production under different experimental conditions, indeed enhances the model’s agreement with observations. When including this mechanism, concentrations of NO(X) near 10^4^ ppm were calculated, mirroring the experimental observations. Note that for this mechanism, we followed Uddi et al.^64^ and considered a gas kinetic rate coefficient. More studies should be performed in the future to assess the validity of this approach. It is also noteworthy that while the agreement in NO(X) density improved, so did the trend associated with the evolution of N_2_O(X) as a function of oxygen content, despite an overestimation of absolute values.

Additionally, we consider the influence of other reactions involving N_2_O, leading to NO(X) production (see the dotted orange line in Figure 13) also with the gas kinetic rate coefficients (reactions **R81**–**R83** in Table 5). When incorporating these additional mechanisms, we note a comparable NO(X) production compared with the previous scenario. However, regarding N_2_O(X) production, it is noteworthy that the trend in N_2_O concentration is now replicated to some extent, with a slight decrease around an oxygen content of 0.01, followed by an increase at high oxygen content.

We further investigated the production of NO(X) under different surface mechanism assumptions. Initially, we eliminate any contribution from wall surfaces in NO(X) production, revealing a significant underestimation of the NO(X) concentration for low oxygen content. However, it is worth noticing that, even when these mechanisms are neglected, the model reproduces the knee-like shape associated with NO(X) production, instilling confidence in the model developed throughout this work. Finally, we explore different values for the recombination energy associated with N(^4^S)_f_ + O(^3^P)_s_ → NO(X) + *S*_*v*_ + *F*_*v*_. While following the discussion of Section 3.3, we have tested the impact of increasing the recombination energy (from 17.5 to 23 kJ/mol). This alteration results in a reduction in NO(X) production, especially at low oxygen content values, which diverges from experimental observations. However, similar to the previous observation, the model retains the ability to reproduce the knee-like shape associated with NO(X) production despite these alterations.

## 5 Conclusions

In conclusion, a comprehensive modeling framework for studying the interactions between volume and surface mechanisms was developed for low-pressure N_2_–O_2_ discharges. This framework establishes a robust foundation for future research focused on analyzing plasma-based NO(X) production while also paving the way for developing reaction mechanisms for N_2_ and N_2_–O_2_ discharges. The inclusion of a mesoscopic description of surface kinetics has afforded valuable insights into the intricate phenomena governing plasma–surface interactions. The model exhibits very good agreement with the experimental data, particularly in terms of NO(X) and N_2_O(X) concentrations/trends, showcasing its efficacy in capturing the evolution of these species in the afterglow of the plasma.

While comparing the experimental data with the modeling outcomes, this study leads to several important conclusions of relevance to low-temperature plasmas dedicated to nitrogen fixation under low pressure conditions. The investigation underscores the significance of atomic nitrogen ground state N(^4^S) and electronically excited states, specifically O(^1^S) and O_2_(b), within the reactor volume. Indeed, the presence of nitrogen atoms emerges as a critical factor in facilitating NO(X) formation. The depletion of nitrogen atoms during the afterglow is directly related to the experimentally observed knee-like NO(X) density as a function of the initial O_2_ concentration in the mixture. Furthermore, the model also reveals how the O_2_(b) state can play a pivotal role in the formation of NO(X) through the reaction O_2_(b) + N(^4^S) → NO(X) + O(^3^P), competing with the N_2_(X, *v* > 12) + O(^3^P) → NO(X) + N(^4^S) process. This competition is particularly relevant in scenarios characterized by high oxygen content (30% – typical of air-like mixtures). Concerning the surface mechanisms, our model highlights the importance of N(^4^S)_f_ + O(^3^P)_s_ → NO(X) + *S*_*v*_ + *F*_*v*_, a process gaining significance at low oxygen content levels. Having atomic nitrogen atoms adsorbed on surfaces that contribute to the formation of NO(X) (under low oxygen content) aligns with the proposal put forth by Ma et al.,^27^ where it was suggested that atomic nitrogen atoms may contribute significantly to NO(X) production through adsorption reactions on Pt surfaces. In line with the incorporation of surface mechanisms, future model improvements should address the impact of atomic nitrogen atoms forming NO(X) species in the presence of a catalyst. This can be achieved by leveraging sticking rate coefficients derived from the transition state theory (as discussed by Ma et al.^27^) while taking into account the mesoscopic description of the reactor surface developed in this work.

This study also underscores the importance of systematic analyses in N_2_–O_2_ discharges and the development of reaction mechanisms, emphasizing the need for validated rate coefficients against the experimental data while various parameters. Future studies aimed at formulating a reaction mechanism for N_2_–O_2_ discharges should address in more detail the (i) variation of reactor wall temperatures while calculating gas temperature, akin to the approach undertaken in Dias et al.^42^ in pure oxygen discharges; (ii) formation of electronically excited states N(^2^D) and N(^2^P) at the reactor wall, which influences the calculated concentration of ground state N(^4^S), and the evolution of electron density through the afterglow; (iii) assumptions related with the formation of N_2_(A) from vibrationally excited N_2_(X, *v*), which influences associative ionization and calculation of self-consistent reduced electric fields in N_2_–O_2_ discharges; and (iv) role of vibrationally excited N_2_(X, *v*) on the formation of NO(X) through the Zeldovich mechanism. Concerning this fourth point, it is essential to highlight that, in this work, a constant value is employed for the rate coefficient associated with N_2_(X, *v* = 13:59) + O(^3^P) → NO(X) + O(^3^P) following Guerra et al.^40^ Recent calculations have demonstrated temperature-dependent rate coefficients for this mechanism.^108^ This aspect should be thoroughly investigated in the next modeling campaigns. This phenomenon also warrants further investigation in future studies dedicated to determining the vibrational temperature of N_2_ in N_2_–O_2_ discharges via detection of the first vibrationally excited levels.

It is worth emphasizing that the model developed in this work stands now as a versatile tool serving as a benchmark for validating volume and surface reaction mechanisms in low-pressure N_2_–O_2_ plasmas. This work establishes the foundation for advancing our comprehension of N_2_–O_2_ plasma systems, presenting new avenues for future research and experimental validation. In particular, it serves as a platform for investigating catalytic and surface mechanisms associated with NO(X) production and other species of relevance for nitrogen fixation in nonthermal plasmas.
